# Context-dependent mechanical reconfiguration is necessary for multifunctional behavior in an intrinsically confined hydrostat

**DOI:** 10.1016/j.isci.2026.117033

**Published:** 2026-08-11

**Authors:** Michael J. Bennington, Stephen M. Rogers, David M. Neustadter, Roger D. Quinn, Gregory P. Sutton, Hillel J. Chiel, Victoria A. Webster-Wood

**Affiliations:** 1 Depts of Mechanical Engineering, Carnegie Mellon University, Pittsburgh, PA 15213, USA; 2 Biomedical Engineering, Carnegie Mellon University, Pittsburgh, PA 15213, USA; 3 Material Science and Engineering, Carnegie Mellon University, Pittsburgh, PA 15213, USA; 4 McGowan Institute for Regenerative Medicine, Carnegie Mellon University, Pittsburgh, PA 15213, USA; 5 Robotics Institute, Carnegie Mellon University, Pittsburgh, PA 15213, USA; 6 School of Natural Sciences, University of Lincoln, Lincoln LN6 7TS, UK; 7 Department of Biology, University of Oxford, Oxford OX1 3EL, UK; 8 Cardiac Success Ltd, Yokneam 2069202, Israel; 9 Department of Biology, Case Western Reserve University, Cleveland, OH 44106, USA; 10 Department of Biomedical Engineering, Case Western Reserve University, Cleveland, OH 44106, USA; 11 Department of Neurosciences, Case Western Reserve University, Cleveland, OH 44106, USA; 12 Department of Mechanical Engineering, Case Western Reserve University, Cleveland, OH 44106, USA

**Keywords:** *Aplysia*, biomechanics, hydrostat, computational modeling

## Abstract

Muscular hydrostats, muscular structures lacking rigid skeletons, are ubiquitous in animals, but how they perform complex actions remains poorly understood. Understanding their function requires uncovering the mechanisms that couple and constrain their deformations. We investigated how mechanical reconfiguration from interacting shape-changing elements in the feeding system (buccal mass) of *Aplysia* facilitates different feeding behaviors. Combining analysis of MRI movies of *Aplysia* feeding with a new biomechanical model, we demonstrate how two context-dependent mechanical reconfigurations produce large protractions in different ways during two key behaviors. During rejection, grasper elongation stretches a protractor muscle, allowing stronger protractions. During biting, the grasper remains compact, requiring additional support from more anterior muscles. The mechanisms integrate shape-changing, bending and conforming muscles, and shifting contacts. We propose two mechanical subclasses of muscular hydrostats, “intrinsically confined” and “unconfined,” that may be morphologically similar but employ different control strategies depending on whether mechanical confines are reliably present.

## Highlights

• Different feeding behaviors in *Aplysia* require distinct neuromechanical mechanisms
• Shape-change and reconfiguration give muscular hydrostats unique mechanical tools
• Bigger structures that bend, not stretch, enhance the efficacy of smaller muscles
• Shared mechanical tools suggest distinct mechanical classes of muscular hydrostats

## Graphical abstract

**Figure:**
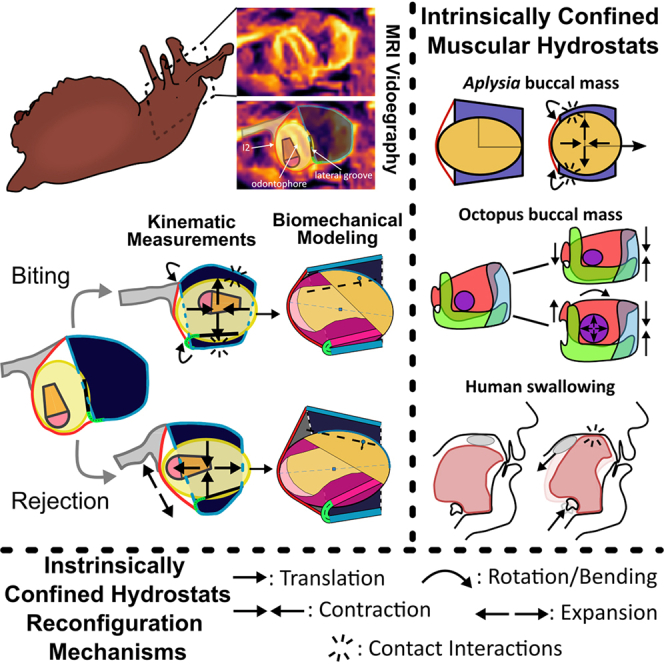

## Teaser

Computational physics; Biomechanics; Applied sciences

## Introduction

Muscular hydrostats are biological structures consisted entirely of muscle and soft connective tissue with no rigid structural elements.^1^ These types of structures are seen in a wide range of phyla, including the tongues of mammals and reptiles, the proboscides of elephants and tapirs, and the bodies and appendages of cephalopods.^1^^,^^2^ These highly flexible and deformable structures allow for a much greater range of movements and behaviors than rigid, jointed skeletal systems. But with this greater flexibility also comes a more challenging control problem for the nervous system. In jointed systems, there are relatively few degrees of freedom (i.e., ways to position and configure the body), often with more muscles than joints, and thus, the nervous system can completely define the position of the body, as well as modulate its properties due to these muscle redundancies.^3^ Muscular hydrostats, in contrast, have a very large number of degrees of freedom. Therefore, they must rely on other ways to constrain their deformations. For example, some cylindrical muscular hydrostats, such as chameleon tongues and octopus arms, rely on networks of cross-helical fibers to couple the length and radius of their structure.^1^^,^^4^^,^^5^ The geometry of these helices can also modulate the mechanical properties (e.g., mechanical advantage) of muscles in the hydrostat.^5^ Muscular articulations in, e.g., the feeding systems of cephalopods and the grasping hooks of flatworms are muscle structures that modulate their shape and stiffness to change the functionality of other muscle groups, allowing a smaller number of muscles to have a greater set of behavioral impacts.^6^ By uncovering mechanisms that allow hydrostats to perform their diverse behavioral repertoire, through careful neuromechanical study of model systems, we can gain insights into the neuromechanical control of soft structures, both biological and engineered.

**Figure 1:**
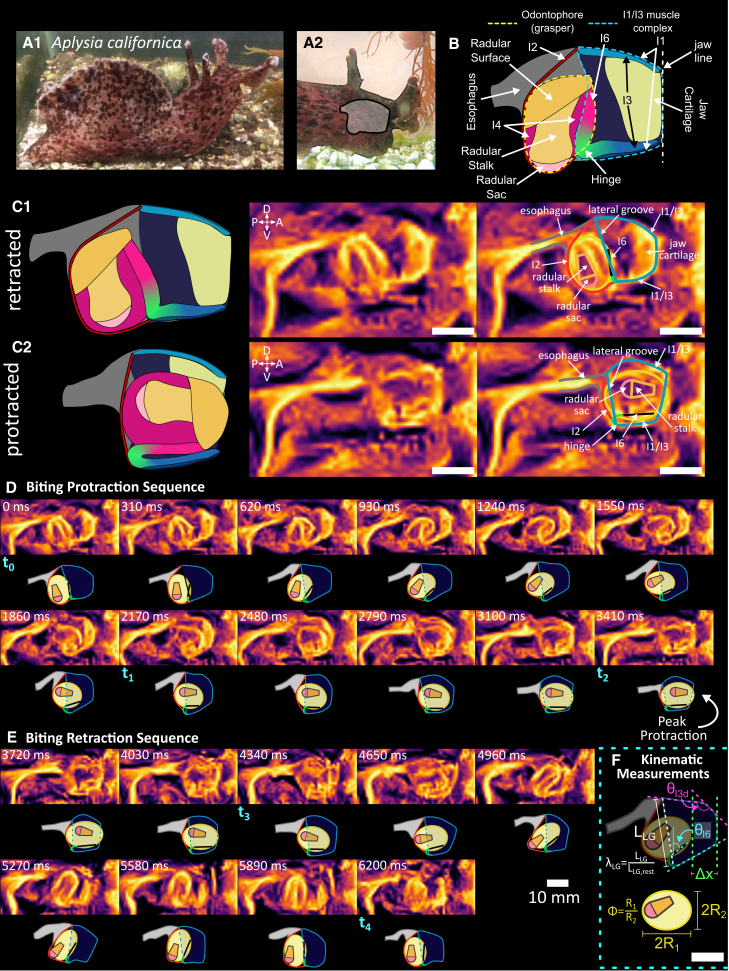
Anatomy and kinematics of the *Aplysia* feeding system (A1) Adult *Aplysia californica* searching for food and (A2) feeding on *Gracilaria* macroalgae. (A1) Photo credit: Dr. Jeffrey P. Gill, (A2) modified with permission from Bennington et al.^7^ under the Creative Commons CC-BY license. Gray highlight shows the location of the feeding structure, the buccal mass (B). (B) An anatomical diagram of a midline sagittal view of a buccal mass. During feeding, the odontophore (the internal grasper of the buccal mass) protracts through the tubelike I3 muscle. In the midsagittal plane, the I3 is visible as two longitudinal elements, but is one continuous structure that runs circumferentially around the buccal mass. The inner wall of the distal I3 is shown in dark blue. The I4 muscle (dark pink structure in the odontophore) is a major portion of the odontophore’s geometry mediolaterally, but is not distinguishable from the general outline of the odontophore in midsagittal views. This muscle is also bilaterally symmetrical, but the half that would be covering the radular stalk has been removed for clarity. The dashed white line shows the jaw line, which is used as the reference for both the translation and rotation measurements. (C) Configuration of the buccal mass (left, anatomical diagram; middle, MRI frames) showing (C1) peak retraction and (C2) peak protraction. (Right) A diagram of the buccal mass was created to highlight key anatomical landmarks for each frame of the MRI video showing a complete biting sequence (D and E). In these diagrams, the yellow ellipse showing the border of the odontophore encompasses the midsagittal boundaries of the I4 muscle and the radular surface. These structures cannot be individually distinguished in the midsagittal MRI views. The same diagrammatic representations of the landmarks are shown in (D and E) for the protraction and retraction portions of the biting sequence, respectively (see STAR Methods). The frames shown in (C1) and (C2) correspond to the 0 ms and 3,410 ms frames, respectively, and are the same between the middle and right portions of the figure. Key frames referred to in the text: t_0_, start of the behavioral cycle; t_1_, peak rotation reached; t_2_, peak translation reached; t_3_, rotation plateau ended; t_4_, end of behavioral cycle. (F) Kinematic measurements were taken using the drawn diagrams for each frame in the sequence. See main text for definitions of variables. All scale bars correspond to 10 mm. See also Figures S1–S3.

The sea hare, *Aplysia californica* (Figure 1A), and its muscular hydrostat feeding system, called the buccal mass (Figure 1B), is an established model system in neuroscience for studying neuromechanical control, multifunctionality, adaptability, and learning across different hierarchical scales.^7^^,^^8^^,^^9^^,^^10^^,^^11^^,^^12^^,^^13^^,^^14^^,^^15^^,^^16^^,^^17^^,^^18^^,^^19^^,^^20^ The study of the buccal mass and its neuromechanics has provided useful insights into the control of muscular hydrostat systems. The feeding system is controlled by approximately 2,000 neurons with large somas that can be readily identified by their location and electrophysiological properties, allowing the behavioral functionalities of individual neurons to be investigated.^21^^,^^22^ Anatomically, the buccal mass primarily consists of two interconnected muscular hydrostat structures.^23^ The inner hydrostat is a ball-like grasper, called the odontophore. The odontophore contains a cartilaginous sheet covered in small teeth, called the radula, which is controlled and deformed by various muscles in the odontophore to grasp onto and release food.^24^^,^^25^^,^^26^ The outer hydrostat structure consists of the I1/I3 muscle complex anteriorly and the I2 muscle, which wraps around the odontophore posteriorly. The I1/I3 complex is a tube-like structure through which the odontophore protracts and retracts during feeding behaviors. It is innervated by several distinct motor pools that allow it to retract the odontophore,^27^ modulate the force applied by the buccal mass to tough food,^9^ and to hold food in place during odontophore protraction.^28^ The I2 serves as the primary protractor muscle of the buccal mass.^29^ Finally, the two hydrostats are interconnected by a structure called the hinge that assists in odontophore retraction and couples odontophore translation and rotation.^15^ The feeding system coordinates these muscles to perform multiple feeding behaviors, two of which are investigated here: biting and rejection.^30^ Biting is a searching behavior in which the odontophore attempts to grasp nearby food. Rejection is a behavior in which inedible objects are pushed back out of the buccal mass by the odontophore. In both behaviors, the buccal mass experiences little load from the environment. In biting, no food is inside the buccal mass to apply loads, and in rejection, the item being expelled is often unanchored or unable to apply compressive forces. Therefore, in these behaviors, the primary mechanical interactions are between different components of the buccal mass itself rather than with the external environment.

Despite much study of the biomechanics and neural circuitry of the *Aplysia* buccal mass, basic questions remain unanswered about how this system operates. One major question relates to how the I2 muscle can protract the odontophore in biting and rejection behaviors.^14^^,^^16^ In both behaviors, the odontophore undergoes large protraction, with the radula extending beyond the jaws of the animal.^16^^,^^31^ When considering only the biomechanical properties of the I2, it appears that I2 should not be able to produce the force needed to protract the odontophore against the passive forces generated by the surrounding tissue.^13^^,^^15^ The low force output from the I2 is the result of two interacting effects: as the odontophore protracts, (1) the I2 becomes shorter and thus moves down on its length-tension curve; and (2) the contact angle between the I2 and the odontophore increases, meaning less of the I2’s force is oriented to protract the odontophore.^14^^,^^16^ Thus, during protraction, the I2 can generate less tension, and any tension that is generated is less effective at pushing the odontophore forward (i.e., its mechanical advantage decreases). Despite this low force output, the protraction still occurs. Studies of rejection behavior suggest that the shape of the odontophore can modulate the force produced by the I2, but that this mechanism cannot explain the protraction observed in biting.^16^ What neuromechanical mechanisms of the buccal mass compensate for the low force output of the protractor muscle and allow for these behaviors to be performed?

In this study, we investigate how the reconfiguration, shape change, and mechanical interactions of muscular hydrostats facilitate complex behaviors. To do this, we examine the kinematics and biomechanics of the *Aplysia* buccal mass during biting and rejection behaviors. We perform a new analysis of MRI video recordings taken of *Aplysia* during biting and rejection behaviors. In this analysis, we identify kinematic differences in the behaviors that suggest context-dependent mechanical interactions in the buccal mass. We then develop and calibrate a hybrid kinematic-kinetic model of the buccal mass. This model captures shape-changing mechanisms in the odontophore and the I1/I3 muscle, geometric and physiological properties of the I2 muscle, and the combined bending and axial stiffness of the hinge structure. Using this model, we conduct computational experiments that demonstrate that the buccal mass requires different combinations of odontophore and I3 configuration and contact interactions for the odontophore to protract in biting and rejection. Finally, we discuss how the mechanisms of reconfiguration identified in the buccal mass may apply to other muscular hydrostats. By identifying shared reconfiguration mechanisms across certain types of muscular hydrostats, we propose that muscular hydrostats may fall into two subclasses—intrinsically confined and unconfined—and that these subclasses may rely on different control schemes because of their available biomechanical tools.

## Results

### Kinematic differences in biting and rejection suggest behavior-specific mechanisms of reconfiguration

*Aplysia* uses its feeding organ, the buccal mass (Figures 1A and 1B), in different ways to ingest and reject food. Biting is an ingestive, searching behavior where animals attempt to grab nearby food.^30^ To do this successfully, the animal must open the radular surface of its odontophore (the internal grasper of the buccal mass, Figure 1B) during the protraction phase (Figure S1), separating the two halves of the surface laterally. This results in the odontophore having a low aspect ratio midsagittally. In contrast, in rejection, previously ingested food is expelled from the buccal mass.^30^ To successfully reject food, the animal must close its radular surface on the object being rejected during the protraction phase, resulting in the odontophore having a higher aspect ratio midsagittally. Previous biomechanical analyses have shown that the I2 protractor muscle weakens as the odontophore protracts, due both to its length/tension properties and its mechanical advantage over the odontophore.^13^^,^^16^ In rejection, this is overcome because the higher aspect ratio of the closed odontophore stretches the I2 muscle more than would an open odontophore.^16^

How does the odontophore (Figure 1B) achieve large protractions during biting and rejection behaviors despite the protractor muscle I2 not being able to produce sufficient force?^13^^,^^16^ Do kinematic differences between biting and rejection point to different mechanisms of mechanical reconfiguration in each behavior? To investigate possible kinematic mechanisms of reconfiguration, we produced a new analysis of *in vivo* midsagittal magnetic resonance imaging (MRI) recordings of biting^31^ (Figure 1; Figure S2) and rejection^16^ (Figure S3) behaviors. Each frame of the behavioral sequence was processed in ImageJ (Fiji^32^) to enhance key anatomical features (see STAR Methods), and schematics for each frame were then created by hand-fitting shape primitives and spline curves to the MRI data (Figures 1C–1E; Figures S2 and S3). Finally, kinematic measurements were taken from the schematics for the full behavioral cycle (Figure 3A). These kinematic measurements (Figure 1F) include the distance from the anterior edge of the odontophore to the anterior edge of the buccal mass (Δ*x*, referred to as translation), and the rotation of the odontophore, measured as the angle (*θ*_*I*6_) between the I6 muscle and the anterior edge of the buccal mass (referred to as the jaw line). I6 forms the anterior edge of the odontophore, and I3 is the anterior tubelike muscle of the buccal mass through which the odontophore protracts and serves to retract the odontophore (Figure S1). We also measured the aspect ratio of the odontophore (Φ) and the configuration of the I3 (odontophore retractor) muscle. Specifically, we measured the stretch (*λ*_*LG*_) of the posterior edge of the I3 (anatomically called the lateral groove, Figure 1C; *λ*_*LG*_ = current length/rest length) and the internal angle between the tangent line at the anterior and posterior edges of the dorsal I3 (*θ*_*I*3*d*_). Key time points in each behavior are identified as t_i_ (biting) and τ_j_ (rejection), with the index ranging from 0 to 4 (note that these are different than the indices reported in Neustadter et al.^31^; see STAR Methods).

In both the biting and the rejection behavior, the radular surface of the odontophore underwent similarly large protractions. In biting, the translation range of motion (ROM) was 0.55 buccal mass lengths (BMLs), and during rejection, it was 0.53 BML. In both behaviors, the tip of the radular surface translates beyond the anterior edge of the buccal mass. For biting, peak protraction beyond the anterior edge was 0.07 BML (t_2_), whereas for rejection, it was 0.09 BML (τ_2_).

However, there were differences between the behaviors regarding the aspect ratio, which has important implications for how the protraction was achieved. At peak protraction, the odontophore in biting was more spherical (aspect ratio, 1.29), corresponding to an opened odontophore (Figure S1). In contrast, the odontophore was more elliptical in rejection (aspect ratio, 1.46), corresponding to a closed odontophore. In addition to extending the long axis of the odontophore itself, this elliptical shape helps to stretch the I2 (odontophore protractor) muscle, enhancing protraction by shifting the I2 muscle higher on its length-tension curve and increasing its mechanical advantage.^14^^,^^16^ But during biting, the I2 is not sufficiently stretched by the more spherical odontophore to exploit this effect.^16^ Thus, another mechanism must be at play.

Further differences were observed in the configuration of the lateral groove. In biting, prior to peak protraction (t_1_ to t_2_), the lateral groove shortened posterior to the odontophore (−45% of its resting length), and θ_I3d_ correspondingly decreased (−17°) (Figure 3A). Together, this suggests that the posterior edge of the I3 was wrapped around the back of the odontophore (Figure 1D). At a similar stage of the cycle in rejection (τ_1_ to τ_2_), this change did not occur (lateral groove shortening, +7%; θ_I3d_ change, +10°, Figure 3A). Additionally, in biting, the initial retraction of the odontophore (t_2_ to t_3_) corresponded with a re-lengthening of the lateral groove and increasing θ_I3d_.

The shortening of the lateral groove and consequent wrapping of the posterior I3 around the rear of the odontophore appears to be a biting-specific kinematic feature, changing how the odontophore and the I3 contact and mechanically interact with each other. We hypothesized that this mechanism will cause protraction of the odontophore, thereby assisting the weakening I2 muscle during biting. However, this mechanism is not seen in rejection, indicating that this reconfiguration may not be necessary for, or may even be detrimental to, rejecting food.

### A hybrid kinetic/kinematic model of the buccal mass reproduces key features of its mechanics

**Figure 2:**
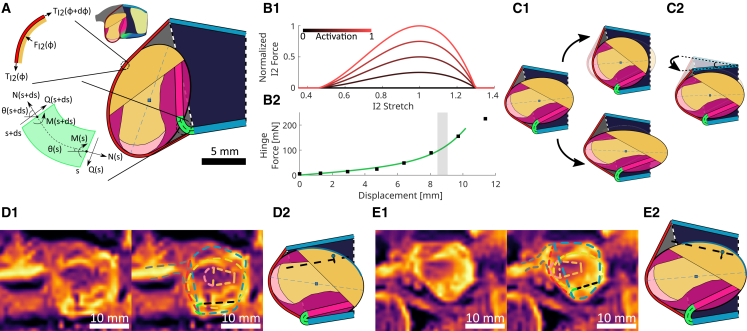
Kinetic/kinematic biomechanical model of the buccal mass (A) Rest geometry of the biomechanical model. The grasper (odontophore) is modeled as a rigid ellipse (magenta, indicating the approximate location of the I4, with yellow radula). It is connected to the I1/I3 lumen (blue trapezoid) by the hinge muscle (green). The I2 protractor muscle (red) wraps conformally around the odontophore and attaches at the lateral groove. The net force and torque from the I2 on the odontophore are found by performing an instantaneous force balance on a small arc of the ellipse and integrating across the full region of contact between the I2 and the odontophore. The hinge muscle is modeled as a linearly elastic, geometrically exact beam. At each position along the beam’s midline, a quasistatic force balance is performed (see STAR Methods). While the I4 muscle is visualized in the odontophore ellipse, it is not explicitly incorporated into the model, only implicitly through the aspect ratio of the ellipse. Scale bar: 5 mm. (B1) The tension in the I2 is modeled using the length-tension relationship reported in Yu et al.^13^ scaled by a normalized activation level. (B2) The axial and bending stiffness of the beam hinge were calibrated to *ex vivo* animal data reported in Sutton et al.^15^ Gray region indicates odontophore displacements observed during biting behaviors (Sutton et al.^15^). (C1 and C2) To investigate the effects of mechanical reconfiguration on odontophore position at peak protraction, (C1) the aspect ratio of the odontophore ellipse and (C2) the stretch of the lateral groove were added as additional kinematic constraints. (C1) and (C2) show results from the model but do not correspond to any particular behavior or configuration observed in the animal. These constraints impact the biomechanical model via contact forces from the I1/I3 (see STAR Methods). The lateral groove stretch is converted to a depression angle of the dorsal I1/I3 muscle as a proxy for the wrapping of the dorsal I3 around the odontophore observed during *in vivo* feeding behaviors (Figure 1). (D and E) MRI frames at peak protraction in (D1, with and without overlay) biting (t_2_) and (E1, with and without overlay) rejection (τ_2_) compared to corresponding frames from the biomechanical model (D2 and E2, respectively). (D) and (E) share the same scale (scale bar: 10 mm). See also Figures S4 and S5.

**Figure 3:**
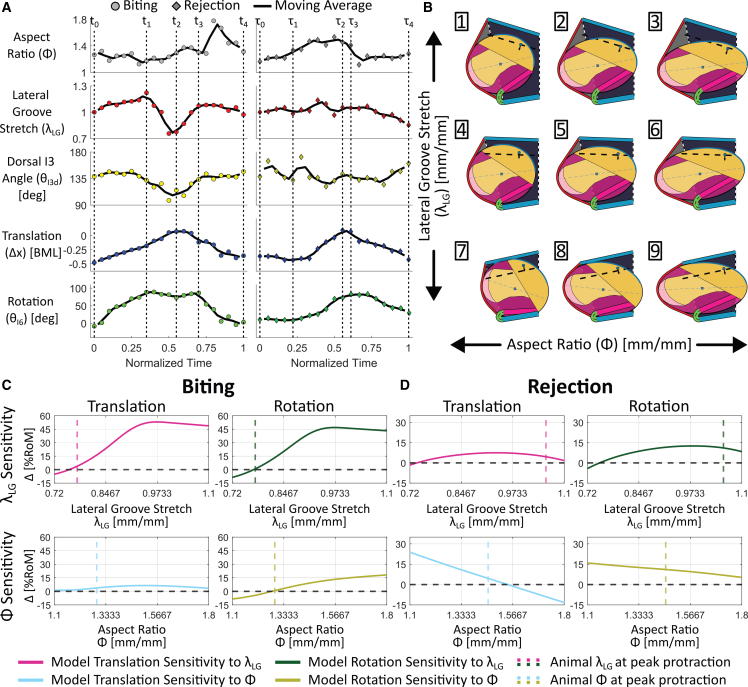
Mechanical reconfiguration of the buccal mass (A) Midsagittal kinematics of the buccal mass during a (left) biting and (right) rejection behavior (see also Figures S2 and S3). Colored circles (diamonds) show data for an individual frame, and the black line shows the two-point moving average of the signal. Vertical dashed lines show concurrent time points in the different kinematic signals (biting: t_0_, cycle starts; t_1_, peak rotation; t_2_, peak translation; t_3_, rotation plateau ended; t_4_, cycle ends. Rejection: τ_0_, cycle starts; τ_1_, rotation plateau ends; τ_2_, peak translation; τ_3_, peak rotation; τ_4_, cycle ends). (B) Model configurations for nine different pairs of aspect ratios (*Φ*) and lateral groove stretches (*λ*_*LG*_) (numbers correspond to the labeled points in Figure S6C). Note that these simulated results from the model do not necessarily correspond to configurations observed in the animal but rather show changes in the system’s configuration due to changes in the kinematic parameters. All configurations here were achieved with an I2 activation of *A*_*I*2_ = 65%. (C and D) Sensitivity of the model translation and rotation at peak protraction to lateral groove shortening (*λ*_*LG*_, top row) and aspect ratio change (*Φ*, bottom row) for biting (C) and rejection (D). The *y* axis for all figures reports the difference (Δ) between the model prediction and observed animal value at peak protraction (for translation or rotation) normalized by the ROM for each behavior. For each figure, one kinematic parameter is held fixed (top, *Φ* fixed; bottom, *λ*_*LG*_ fixed) at the value observed in the animal at peak protraction, and the other is varied to determine the effect of changing this parameter on the translation and rotation of the odontophore. Vertical dashed lines show the observed value of the varied parameter in the animal at peak protraction. The horizontal dashed line shows zero difference for reference. The steepness of the difference curve in the vicinity of the vertical dashed line indicates how sensitive the system is to changes in each kinematic parameter near peak protraction. Here, a steeper curve (with a positive or negative slope) indicates greater sensitivity. For biting simulations, *A*_*I*2_ = 15%, and for rejection, *A*_*I*2_ = 90% based on the results of the model validation. Each curve in (C and D) is a 1D cross-section of the 2D contour plots shown in Figures S6 and S7. For a complete view of the sensitivity of translation and rotation to lateral groove stretch and aspect ratio across the kinematic configuration space at different I2 activations, see Figures S6 and S7. Note that (C and D) use different vertical scales. The smaller scale for the rejection plots was chosen to better show the difference curves for rejection, and it reflects the overall decreased sensitivity to both lateral groove stretch and aspect ratio changes for the rejection behaviors. See also Figures S2–S7.

To investigate this hypothesis of behavior-specific reconfiguration, we developed a hybrid kinetic/kinematic biomechanical model of the buccal mass (Figure 2A). For full details, see STAR Methods. Briefly, in this model, user-controlled kinematic degrees of freedom interact with a quasistatic^7^^,^^33^ midsagittal biomechanical model of the buccal mass through elastic contact forces. In the kinetic model, the I2 muscle, whose length-tension properties are modified from Yu et al.,^13^ wraps conformally around and applies force to an elliptical odontophore. The odontophore is connected to the rest of the buccal mass by the hinge (the junction between muscles I6 and ventral I3; Figure 1B, green), modeled as a beam element.^34^ In the kinematic model (Figure 2C), the aspect ratio of the odontophore (Φ, ranging from 1.1 to 1.8) corresponds to how open (smaller Φ) or closed (larger Φ) the odontophore is. Additionally, the lateral groove stretch (*λ*_*LG*_, ranging from 0.72 to 1.1) sets the shape of the posterior I3. The range of kinematic values was chosen to span the range observed *in vivo* (Figure 3A). Elastic contact forces from the I3 muscle allow these kinematic subsystems to mechanically interact with the kinetic model. As a first approximation, we did not incorporate the wrapping of the I3 (see Discussion). Anatomical and biomechanical properties of the model were calibrated to existing animal data^15^^,^^31^ and models^13^ (Figures 2B and S12). To ensure the model would provide meaningful insights into the reconfiguration, we validated its ability to capture key biomechanical and behavioral features of the buccal mass.

We first validated that the model reproduced the hinge’s force response across the odontophore’s full ROM. Prior models utilizing spring-like muscles either failed to capture the anatomy of the hinge^14^^,^^15^^,^^17^ or failed to capture the low-displacement force response of the hinge.^7^ Re-examination of the hinge’s anatomy (Figure 1B) led us to hypothesize that the shortcomings were due to a lack of bending stiffness in the modeled hinge.^7^ Thus, we modeled the hinge as a beam, combining axial and bending stiffness in a single mechanical element. The beam model of the hinge well approximated *ex vivo* force-displacement responses^15^ (root mean-squared error of 3.86 mN across all behaviorally relevant displacements compared to expected force ∼100 mN at peak protraction) within behaviorally relevant displacements (Figure 2B). A sensitivity analysis showed that the hinge forces at low odontophore displacements are due solely to the bending stiffness of the beam, with the axial stiffness contributing only at large displacements (Figure S4). Together, these results ensure we can predict behaviorally relevant forces from the hinge and support our hypothesis about the role of bending stiffness in the hinge.

Next, we validated that the buccal mass model could achieve the same peak protraction configurations as observed in the MRI data. By activating the I2 (odontophore protractor) muscle and applying the odontophore aspect ratio (Φ) and lateral groove stretch (*λ*_*LG*_) observed in the MRI data (Figure 3A), the model successfully reproduces the peak protraction configuration for both biting (Figure 2D) and rejection (Figure 2E) behaviors. The model also achieves a similar level of peak translation to the animal data (biting, model peak translation of 0.051 BML, error of 3.8% of the animal’s behavioral ROM; rejection, model peak translation of 0.070 BML, error of 4.6% ROM). The model approximates the rotation at peak protraction for biting well (level of rotation, 72.6°; error of 0.76% ROM), but underestimates the rotation in rejection (level of rotation, 64.7°; error of 11.2% ROM). This may be due to deformation observed in the ventral I3 in the MRI data (Figures 2D and 2E) that is not modeled with the rigid bar approximation of the ventral I3.

Finally, although the level of protraction was similar in both behaviors, the biting configuration in the model could be achieved for a lower activation of the I2 muscle (*A*_*I*2_ ≈ 15%) than was required for rejection (*A*_*I*2_ ≈ 90%) (Figure S5). A greater I2 activation would take longer to achieve and to relax in the animal, leading to longer overall behaviors. This agrees with and helps to explain observations in animals that rejection behaviors tend to take longer than biting behaviors.^19^^,^^35^

### The buccal mass utilizes behavior-dependent reconfiguration to protract the odontophore

From our kinematic analysis of the buccal mass, we observed that the lateral groove shortens during the late protraction phase of the biting behavior (t_1_ to t_2_), but not during the rejection behavior (τ_1_ to τ_2_) (Figure 3A). Additionally, the behavior being performed dictates the odontophore’s aspect ratio.^16^^,^^31^ For biting, the odontophore must be open (lower aspect ratio) during protraction to successfully grab food. In rejection, it must be closed (higher aspect ratio) during protraction to effectively push food out of the buccal mass. Thus, to investigate why lateral groove shortening is used in biting but not rejection, it is necessary to understand how these two mechanisms interact. To investigate the sensitivity of the buccal mass system to these interactions at peak protraction, we performed computational experiments with the model to probe the full kinematic parameter space (Figures S6 and S7). We conducted simulations in which the odontophore was protracted under the action of the I2 muscle, and the model’s kinematic parameters (*λ*_*LG*_ and Φ) were systematically varied while measuring translation (Δ*x*) and rotation (*θ*_*I*6_) of the odontophore. Contour plots of translation and rotation at peak protraction for biting (Figure S6) and rejection (Figure S7) across the kinematic parameter space were generated. Finally, cross sections of the contour plots were taken around physiologically relevant configurations to investigate the system’s sensitivity to each parameter at peak protraction (Figures 3C and 3D).

When the odontophore is open (as in biting, aspect ratio ∼1.3), the lateral groove shortening leads to odontophore protraction for nearly all levels of I2 activation (Figures S5 and S6). Plotting the difference between the translation achieved by the model for a given lateral groove stretch and the level achieved by the animal at peak protraction reveals the system’s sensitivity to lateral groove shortening (Figure 3C, top). Specifically, the sensitivity is seen in the steepness of the difference curves near the lateral groove stretch observed in the animal at peak protraction. Conversely, the model is largely insensitive to odontophore shape changes when the lateral groove shortens in biting-like configurations, shown in the much flatter difference curve (Figure 3C, bottom). Additionally, even for large activations of the I2 (Figure S6), if the odontophore is open, it cannot reach the translation or rotation observed in the animal without lateral groove shortening. This suggests that with an open odontophore, as in biting behaviors, the buccal mass will require lateral groove shortening to protract the odontophore far enough forward to effectively grab food. Lateral groove shortening is also sufficient to protract the odontophore over a wide range of I2 activations, making it a versatile mechanism for biting behaviors. This supports our hypothesis that lateral groove shortening assists the I2 in protracting the odontophore during biting behaviors. But why is this mechanism only utilized in biting?

When the odontophore is closed (as in rejection, aspect ratio ∼1.5), sensitivity to shortening of the lateral groove at peak protraction decreases. This effect can be seen in the flatness of the difference curves in Figure 3D (top) and in the contour plot isocurves bending upwards away from the horizontal axis (Figure S7). The decreasing sensitivity is especially pronounced as the protractor muscle (I2) activation increases. In contrast, the system is more sensitive to changes in aspect ratio (which will change the length of the I2 muscle) (Figure 3D, bottom). Finally, even at the resting lateral groove length (*λ*_*LG*_ = 1), only I2 activation is needed to achieve large odontophore translations if the odontophore has a high aspect ratio (Figure S7). This suggests the buccal mass does not need lateral groove shortening to achieve successful protraction of a closed odontophore, as seen in rejection, relying instead on the high aspect ratio of the odontophore to stretch the I2 muscle.^16^

## Discussion

### Bending appears to play a key role in the mechanical reconfiguration of the buccal mass

Previous analyses of the buccal mass and many other biomechanical systems have focused on the stretching of tissue and the bulging of structures.^7^^,^^14^^,^^15^^,^^16^^,^^17^ However, in this work, we show two key features that rely not on the stretching of tissue along its length, but rather on its bending. In fact, the ability of structures to bend without stretching is critical to their functionality in this system and provides ways for smaller muscles to play an outsized role in the biomechanics of feeding.

The first bending structure is the hinge, connecting the odontophore to the I3, and modeled here as a beam. Previous models of the buccal mass have treated the hinge as an axial spring acting to resist odontophore translation.^7^^,^^14^^,^^15^^,^^16^^,^^17^ During calibration of this model, we found that at low odontophore displacements, the force from the hinge was only sensitive to the bending stiffness of the hinge (Figure S4). This implies that, for small displacements, the hinge is only bending and not stretching (the kinematics of the beam in these computational experiments also shows bending alone, Figure S4). Only at large displacements did the hinge begin to stretch along its length. This bending-dominated kinematics has two effects: (1) the translation and rotation of the odontophore can be coupled in ways not available to purely spring-driven systems, and (2) large translations of the odontophore can be achieved for small input forces without requiring the resisting structure to be weak. Smaller input forces are sufficient because of the mechanical advantage afforded by the moment arm of the load being applied to bend the tissue (Figure S11).

The second bending structure in this system is the dorsal edge of the I3. For simplicity, we modeled the effect of this wrapping as depression of the dorsal I3. However, in the MRI (Figure 1), we see that the dorsal I3 conforms to the shape of the odontophore, that is, it bends around the perimeter of the odontophore. This bending and wrapping play key roles in I3’s functionality. First, by wrapping around the back of the odontophore, the posterior contact forces point even more anteriorly, helping to amplify the effectiveness of I3 as a protractor assistant. This would mean that the rigid I3 approximation used in this model may be a conservative estimate of the effectiveness of I3 as a protractor assistant. Second, the wrapping and subsequent contact with the odontophore allow the dorsal surface of the I3 to act as a Class II lever (Figure S8), with the load acting to protract the odontophore and the effort being supplied by I2. This configuration allows the I3 to serve as a force multiplier for I2 and provides a more advantageous moment arm to further bend the hinge. Traditionally, a lever requires a fixed pivot point (the fulcrum). In the model, the dorsal I3 can pivot about its anterior endpoint (Figure 2C). However, bending allows for structures to function like a Class II lever but without a true pivot joint, which could not be achieved by a continuous structure like a hydrostat.

The bending of the dorsal I3 may be the result of different interacting components. First, the I2 is well-positioned to apply a bending moment to the I3 as it applies force at a position maximally distant from its base. Additionally, in biting behaviors where this bending is seen, the shape and position of the odontophore means that I2 is positioned more perpendicularly to the average direction of the I3, therefore increasing the resultant moment it generates for any level of activation. Finally, the bending could be influenced by the passive mechanics of the I3 itself and the lateral contact with the odontophore. As the odontophore opens, it will widen mediolaterally, pushing the lateral walls of the I3 outward. This will tend to cause the dorsal and ventral extremes of the I3 to move medially, which in the midsagittal plane would manifest as a bending of the I3. Future studies to investigate these mechanisms will require a 3D odontophore and I3 model and additional coronal kinematic information.

### Lateral groove shortening may be detrimental to the effectiveness of rejection behaviors

The model demonstrated that the buccal mass can protract the odontophore under I2 activation alone when the odontophore is in a closed configuration (as it is in rejection behaviors). Additionally, the shortening of the lateral groove, which is necessary in biting behaviors, is ineffective in protracting the odontophore when the radular surface is closed. So, mechanically, the mechanism isn’t necessary for complete protraction of the odontophore in rejection. From a behavioral perspective, the shortening of the lateral groove and subsequent posterior wrapping of I3 may be disadvantageous for rejection. As the lateral groove shortens, the contact between the odontophore and the I3 increases. This increases the contact force that assists in protraction, but it could also pinch the item being rejected between the odontophore and the posterior I3. This would hold that item in place and make it more difficult for the odontophore to expel it from the buccal mass. This is not a concern in biting behaviors, as biting is a searching behavior performed when there is no food in the buccal mass. Therefore, this mechanism may not be used in rejection both because it is not necessary for the protraction of a closed odontophore and because it may lead to decreased performance in expelling inedible objects.

### Contact interactions guide the kinematics of the buccal mass feeding cycle

From our modeling efforts, we found that the changing contact interactions between the odontophore and the I3 were critical for achieving the correct configurations at peak protraction. Anterior contact between the odontophore and the dorsal lumen of the I3 provided a way for the odontophore to rotate forward and pitch the radular surface down. The changing magnitude and direction of the contact force from I3 also contributed to odontophore protraction for biting-like configurations. Without these contact interactions, the odontophore would not be positioned appropriately at peak protraction to grasp food and successfully feed. Finally, the magnitudes of the contact forces were modulated by other structures (e.g., by the aspect ratio of the odontophore). This modulation allows the contact interactions to play different, context-dependent roles in behavior.

### Shared mechanical reconfiguration mechanisms suggest there may be subclasses of muscular hydrostats

Muscular hydrostats, with their continually deforming structures, simultaneously offer a remarkable degree of mechanical and behavioral flexibility and present a daunting control task.^1^^,^^36^^,^^37^^,^^38^ Despite this difficulty, animals with muscular hydrostats routinely solve complex control problems to perform flexible behaviors. In other biomechanical systems, the mechanics of the body can offload neural control tasks, thus simplifying the task of descending control.^39^ For example, muscle preflexes allow the body to respond to and correct for mechanical perturbations faster than the nervous system can respond.^40^^,^^41^ The mechanically mediated stretch activation observed in insect flight muscle^42^^,^^43^ allows insect wings to oscillate faster than could be obtained through neural stimulation. Similar stretch activation mechanisms in cardiac tissue allow the heart to modulate its force production to withstand changing preloads and afterloads without direct neural control.^44^ Collectively, the role that the body’s mechanics play in its own control is often referred to as morphological computation or embodied intelligence.^45^

**Figure 4:**
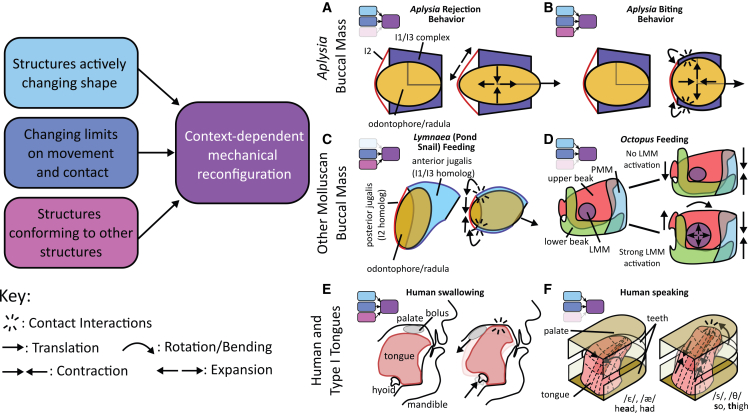
Mechanical reconfiguration facilitates behaviors in a variety of intrinsically confined hydrostat systems Combinations of the active shape change of internal structures (cyan), changes to the movement constraints and contact interaction (blue), and bending and conforming of structures (magenta) allow intrinsically confined hydrostats to mechanically reconfigure their neuromusculature (purple) to perform various behaviors. This can be seen in various systems across various species. As discussed here, the *Aplysia* buccal mass uses combinations of these mechanisms in (A) biting and (B) rejection behaviors to protract the buccal mass. (C) The pond snail, *Lymnaea*, has a morphologically similar buccal mass to *Aplysia*, but its I1/I3 homolog, the anterior jugalis, sits further posterior to the odontophore,^46^ meaning it may more readily rely on the bending of the anterior jugalis and contact interactions during protraction. (D) The octopus and, more broadly, cephalopod buccal masses contain a beak that lacks a fixed articulation. Instead, by activating the lateral mandibular muscle (LMM), the buccal mass can create a stiff rotation point and may shift the function of the posterior mandibular muscle (PMM) from compressing the buccal mass to opening the beak.^47^^,^^48^ (E) The human tongue (and other Type I tongues^49^) sits within the skull and makes use of contact with the hard palate to push food from the oral cavity into the pharynx.^38^^,^^50^ (F) Additionally, by changing how the tongue interacts with the palate and teeth, while maintaining the same internal shape, humans can produce various vowel and consonant sounds.^51^^,^^52^^,^^53^ This use of contact with the palate and teeth is known in the phonetics community as “bracing.” Here, by creating a groove in the middle of the tongue, the phonemes/ε/ and /æ/can be produced. By raising the tongue and creating palatal contact while maintaining that groove, these vowels shift to the fricative consonants /s/ and /θ/.^51^ Small insets show which of the mechanical configurations are used in each behavior.

What mechanisms of morphological computation may be at play in muscular hydrostats? The *Aplysia* buccal mass suggests three such mechanisms (Figures 4A and 4B). During rejection behaviors, the odontophore actively changes shape by closing the radular surface and increasing its aspect ratio. The resulting stretch of I2 increases the force applied by the odontophore protractor (I2) muscle to the odontophore’s posterior edge, enhancing protraction beyond what neural activation of the I2 alone could produce.^16^ During biting, the odontophore changes to its open, more spherical configuration, and the posterior edge of I3 conforms to the odontophore (Figure 1). The conforming I3 interacts with the odontophore shape change to modulate the contact interaction between the odontophore and the dorsal I3, aiding protraction. Thus, during these two behaviors, the buccal mass uses combinations of (1) actively shape-changing structures, (2) changing mechanical advantage and contact interactions between interacting bodies, and (3) the conformability of structures to other structures.

We believe these mechanisms may be applicable to understanding the mechanics and control of a mechanical subclass of hydrostats we will refer to as “intrinsically confined hydrostats” (Figure 4). These intrinsically confined hydrostats, e.g., the buccal masses of other mollusks^46^^,^^47^^,^^48^ (Figures 4C and 4D) and the intra-oral (Type I) tongues of humans and other vertebrates^38^^,^^49^^,^^52^ (Figures 4E and 4F), operate within the confines of other biological structures. These confines limit the ROM of these hydrostats but also provide them with new opportunities for mechanical interaction (e.g., luminal muscle conformation in *Aplysia* and *Lymnaea*, Figures 4A–4C; muscular articulations^48^ in cephalopods; Figure 4D; palatal contact in human tongues; Figures 4E and 4F). A key feature of the subclass is that, in these hydrostats, the intrinsic mechanical confines are consistently present and thus can be reliably used to simplify or constrain the neural control through morphological computation. The odontophore of the *Aplysia* buccal mass always operates within the lumen of the I3; the human tongue predominantly operates within the oral cavity. Because the mechanical interactions (both constraints and opportunities) imposed by the hydrostat’s confines are always present during behavior and co-evolved with the nervous system, the neural circuitry controlling these muscular hydrostats will likely reflect the peripheral biomechanics and the morphological computations it performs.

The mechanical environment of intrinsically confined hydrostats contrasts with “unconfined hydrostats,” such as cephalopod arms and elephant trunks. Beyond their attachment to the rest of the body (an extrinsic constraint at one boundary), unconfined hydrostats are less constricted and predominantly interact with the external environment only. Therefore, they may not be able to take advantage of the same control mechanisms that intrinsically confined hydrostats do. This sub-classification does not imply that unconfined hydrostats experience no constraints on their movements; rather, they must rely on constraints imposed by their internal anatomy and physiology or transient environmental interactions, rather than those imposed continuously by their surroundings. As mentioned previously, various hydrostats rely on architectures of cross-helical fibers to couple longitudinal and radial deformations.^1^^,^^5^ Recent studies of the octopus arm demonstrated regional specialization of the anatomy to localize different deformation modes (e.g., bending and stretching).^54^ Additionally, differences in the length-tension relationship and the resting position of the muscles along this curve between the longitudinal and transverse muscles of the octopus arm constrain how these muscles contribute to deformation and, therefore, behavior.^55^ Thus, unconfined hydrostats can simplify the dimensionality of their control using internal anatomical and physiological constraints, but their nervous systems cannot depend on predictable contact and conformation with other structures in the ways afforded to intrinsically confined hydrostats.

Differences between the mechanical environments of the two subclasses of muscular hydrostats suggest different control strategies. Additionally, hydrostats of the same mechanical subclass may have more similar control mechanisms to each other than to more homologous structures in the opposite subclass. Evidence for intra-class similarity in control strategy can be seen in the behaviors of elephant trunks and octopus arms (two unconfined hydrostats). Despite being very evolutionarily distant animals, both systems have converged on a similar control simplification. Both elephants and cephalopods control their unconfined hydrostat biomechanics through a system of chained-together motor/motion primitives in which simpler actions (e.g., elongation, bending, stiffening) can be independently controlled and chained together to form more complex behaviors.^36^^,^^37^ Both systems also utilize “pseudo-” or “temporary-joints” to achieve precise point-to-point movements.^36^^,^^37^ This convergent evolution to a similar control strategy may be caused by the shared mechanics between these systems. A corollary to this argument would be that we would expect to see divergent control strategies between muscular hydrostats with different mechanics, even if the gross functionality is the same. For example, the long and reaching extra-oral (Type II) tongues of, e.g., pangolins^49^^,^^56^ may have a more similar control scheme to that of an octopus arm than to the intra-oral control of the human tongue, even though both tongues serve to ingest food.

Interesting model systems to explore the control schemes associated with these two subclasses could be hydrostats that operate both within and without confining structures. These could include the Type I tongues that are also used for food searching and object grasping, like those of cows.^49^ These tongues both operate within the oral cavity for food mastication and swallowing, and external to it for grasping and tool use.^57^ This subclass hypothesis would suggest that the control schemes used in reaching and grabbing tasks may be more like unconfined hydrostat control, whereas those used for masticating and swallowing would more closely resemble intrinsically confined hydrostat control. More behavioral and computational neuromechanical investigations are needed to thoroughly understand these systems.

### Limitations of the study

The kinematic analysis presented in this study, and its comparison to model predictions, were based on a single representative behavior, and thus, statistical comparisons cannot be made. We believe this is appropriate in the case of the kinematic analysis, as the dataset from which the data were drawn contained data from eight animals across many behaviors (71 bites, 174 swallows, 10 rejections). Previous analyses of these data^31^ have shown that the kinematics were repeatable when time-normalized to the cycle length. Qualitatively similar observations of biting and swallowing protraction and retraction have also been made in external views of animals (such as in Figure S1), in transilluminated juveniles,^58^ and in reduced buccal mass preparations.^28^ Here, the representative biting behavior was chosen as one that most clearly showed the configuration of the system throughout the feeding cycle and in which the freely behaving animal moved mediolaterally the least. For rejection, we were limited to only one cycle in which the animal’s entire head did not move during the cycle. During rejection, animals often also retract their head to move away from the rejected item, making the MRI experiments more difficult for this behavior (unpublished observations, H.J.C.).

Additionally, it has been demonstrated that analyses of, or models fit to, inter-animal averaged data or averaged parameter sets may not be representative of any individual in a population.^15^^,^^59^^,^^60^^,^^61^ Thus, detailed analysis of representative individuals (as conducted here) may be more appropriate. This does leave open the question: “how representative are these mechanisms to every biting and rejection behavior within an individual or across individuals?” Given the coupled nature of the system (enforced by its anatomy) and the level of protraction achieved in the model without these mechanisms, we believe that these mechanisms are widely shared across different animals. To directly answer this question, it would be best to obtain coupled datasets for individual animals that provide information at least for anatomy and behavior, and ideally for mechanical properties. With such a dataset, individualized models of animals could be constructed, and the coupling between behavior and mechanics could be investigated in a representative population. However, with current methods and existing legacy datasets, this remains a daunting task for such internal measurements of soft-bodied structures.

Finally, in the current analysis of the buccal mass, we only investigated the impacts of mechanical interactions of the buccal mass with itself (e.g., the wrapping and contact between I3 and the odontophore). This simplification was appropriate for the behaviors studied here (biting and rejection) because in these behaviors, there is little mechanical loading from the environment. However, the mechanical forces from foods being ingested are an important factor when studying feeding behaviors. Previous studies of *Aplysia* feeding have shown that swallowing behaviors are modified by the presence of increased mechanical loads from tough food.^9^^,^^62^ More broadly, feeding with tongues (or tongue-like structures) will be impacted both by the mechanics and confines of the tongue and the material properties of the food being ingested. For example, the temporal dynamics of swallowing in humans change when consuming liquids of varying viscosity.^63^ Future investigations of feeding with intrinsically confined and unconfined hydrostats should study how these external mechanical forces are accommodated by the nervous system and how they impact the mechanisms used by the peripheral biomechanics.

## Resource availability

### Lead contact

Requests for further information and resources should be directed to and will be fulfilled by the lead contact, Victoria A. Webster-Wood (vwebster@andrew.cmu.edu).

### Materials availability

No new reagents or materials were generated in this work.

### Data and code availability

• All model code and scripts to conduct computational studies and data analysis are available through GitHub. An archived version of the code is available on Zenodo (https://doi.org/10.5281/zenodo.21037627).
• All experimental and computational data generated during this study is available in the same GitHub and Zenodo archive as model code.
• Any additional information required to reanalyze the data reported will be shared by the lead contact upon request.

## Acknowledgments

This work was supported by NSF DBI2015317 as part of the NSF/CIHR/DFG/FRQ/UKRI-MRC Next Generation Networks for Neuroscience Program. M.J.B. was supported by the NSF Graduate Research Fellowship Program under grant no. DGE2140739 and the Carnegie Mellon University College of Engineering Presidential Fellowship. Any opinions, findings, conclusions, or recommendations expressed in this material are those of the authors and do not necessarily reflect the views of the National Science Foundation. S.M.R. and G.P.S. were also supported by the UK Medical Research Council (MRC) as part of the NeuroNEXT program (MR/T046619/1), and G.P.S. received funding from the Royal Society (URF∖R∖191025). The authors would like to thank Dr. Jeffrey Gill for the use of his photographs of *Aplysia*.

## Author contributions

Conceptualization, M.J.B., S.M.R., G.P.S., H.J.C., and V.A.W.-W.; data curation, M.J.B., S.M.R., and D.M.N.; formal analysis, M.J.B.; funding acquisition, G.P.S., R.D.Q., H.J.C., and V.A.W.-W.; methodology, M.J.B., S.M.R., D.M.N., H.J.C., and V.A.W.-W.; project administration, M.J.B. and V.A.W.-W.; resources, V.A.W.-W.; software, M.J.B.; supervision, H.J.C. and V.A.W.-W.; visualization, M.J.B. and S.M.R.; writing – original draft, M.J.B. and V.A.W.-W.; writing – review and editing, M.J.B., S.M.R., D.M.N., R.D.Q., G.P.S., H.J.C., and V.A.W.-W.

## Declaration of interests

The authors have no conflicting interests to declare.

## Declaration of generative AI and AI-assisted technologies in the writing process

During the preparation of this work, the authors used Grammarly to improve grammar, spelling, and clarity. No new text was generated using the tools. After using this tool or service, the authors reviewed and edited the content as needed and take full responsibility for the content of the publication.

## STAR★Methods

### Key resources table

**Table:** 

REAGENT or RESOURCE	SOURCE	IDENTIFIER
**Biological samples**		

*Aplysia californica*	Marinus Scientific, Long Beach, CA, USA	N/A

**Deposited data**		

Raw and analyzed data (including data generated by the model)	This paper	GitHub Repository (archived doi: https://doi.org/10.5281/zenodo.21037627)

**Experimental Models: Organisms/strain**		

Adult California sea hares (*Aplysia californica*)	Marinus Scientific, Long Beach, CA, USA	N/A

**Software and algorithms**		

MATLAB r2024a	MathWorks	MathWorks
WebPlotDigitizer v4	Ankit Rohatgi^64^	WebPlotDigitizer
ImageJ Fiji	ImageJ	ImageJ Fiji
Inkscape	Inkscape	Inkscape
Finite Difference Coefficients Calculator	Cameron Taylor	Finite Difference Coefficient Calculator

### Experimental model and study participant details

All animal kinematic, anatomical, and mechanical data used in this study are from prior experiments (Sutton et al.^15^; Novakovic et al.^16^; Neustadter et al.^31^). One *Aplysia californica* specimen was used to obtain external videos of biting behavior (Figure S1). This animal was wild caught in Long Beach, CA, USA by Marinus Scientific, and it was kept in a 40-gal artificial seawater (Instant Ocean) aquarium at Carnegie Mellon University (15°C–16°C, specific gravity 1.023–1.03). All animals were beyond the size of sexual maturity and are hermaphroditic, so no sex-dependent considerations were taken.

### Method details

#### Animal anatomy and kinematics imaging

To investigate the configurations of the *Aplysia* buccal mass during biting and rejection behaviors, particularly at peak protraction, we analyzed prior MRI video datasets of *in vivo* feeding behaviors. These MRI videos were originally collected as part of the studies reported in Neustadter et al.^31^ and Novakovic et al.^16^ For complete details of the MRI procedure, we refer the reader to Neustadter et al.^65^ Briefly, MRI videos were recorded of the midsagittal plane of the buccal mass during feeding behaviors. Behaviors were elicited using various stimuli, including strips of dried seaweed and noodles flavored with seaweed. All frames took 155 ms to obtain and were separated by 310 ms. One biting sequence (sequence 7521_S4, frames 31–51) and one rejection cycle (sequence 3229_S1, frames 85–103) were chosen to analyze. The biting cycle was chosen as it had not been previously analyzed and a complete feeding cycle could be observed without significant mediolateral motion. The rejection cycle is the same cycle as was investigated in Novakovic et al.^16^, as this was the only sequence in the dataset with sufficiently low mediolateral motion or parallax.^16^ All MRI frames were post-processed in ImageJ Fiji^32^ (Figure S9). Specifically, the region surrounding the buccal mass was cropped (Figure S9A), and local contrast enhancement (CLAHE, blocksize = 20, histogram = 256, maximum slope = 3, Fiji^32^) was performed (Figure S9B). Then the images were upsampled by a factor of 3 using bicubic interpolation^66^ (Figure S9C) and false-colored using the inverted ‘mpi-inferno’ look-up table (Figure S9D). For the images used here, we observed that the order of interpolation and contrast enhancement did not make a noticeable difference.^66^ The frame showing peak protraction in the biting sequence was then identified as the first frame where the odontophore reaches its furthest level of translation beyond the anterior edge of the buccal mass. Using the MRI frames and the high-resolution lateral view of a bisected buccal mass reported in Neustadter et al.,^31^ anatomical schematic drawings were created for the rest, retracted, and protracted configurations of the buccal mass using Inkscape (The Inkscape Project, Boston, MA, USA). Additionally, for each frame, schematic diagrams were drawn using Inkscape, depicting the radular stalk, the odontophore outline (drawn as an ellipse), the outline of the I1/I3 complex, the locations of the I6 and hinge muscles connecting to the I1/I3 complex, and the esophagus. Structures were identified in the MRI frames with reference to anatomical images and by comparing sequential frames to differentiate consistent structures from background objects. These MRI frames, in combination with the schematic diagrams, were used for qualitative analysis of the kinematics and to compare with configurations produced by the model. Kinematic measurements were also taken from the schematic diagrams for the full feeding cycle (see main manuscript Figure 1F). These measurements include.

1) the distance from the front of the odontophore to the jaw line (“translation”, Δ*x*),
2) the angle between the I6 muscle and the anterior edge of the tubelike I3 muscle (“rotation”, *θ*_*I*6_),
3) the length of the lateral groove (and its stretch, *λ*_*LG*_ = lateral groove length/lateral groove rest length),
4) the aspect ratio of the odontophore (Φ), and
5) the change in tangent angle between the anterior and posterior edges of the dorsal I3 (*θ*_*I*3*d*_).

These measurements were made using lines drawn on the schematic diagrams in Inkscape and rescaled from Inkscape units to millimeters using the scale of the MRI images (see Figure S2 for measurements in biting; Figure S3 for measurements in rejection).

Key timepoints in the behaviors were then identified from the time series translation and rotation kinematics data (labeled t_i_ (i ∈ [0,4]) for biting and τ_j_ (j ∈ [0,4]) for rejection). The time points are different than the periods identified in Neustadter et al.^31^ (labeled t_1_ through t_4_ in that paper). These time points include the start (t_0_, τ_0_) and end (t_4_, τ_4_) of the cycle, and the time of peak translation (t_2_, τ_2_) and peak rotation (t_1_, τ_3_). The final identified point is the end of an identified rotation plateau (t_3_, τ_1_). For biting, this plateau occurred when the odontophore is protracted, but for rejection, it occurs when the odontophore is retracted. We identified these time points based on the rotation and translation kinematics because these values characterize the gross configuration of the system and are the dependent variables of our model. The other kinematic variables relate to the independent control variables in the model. By identifying key points in the dependent variable time series, we can better identify how these control variables may impact the configuration of the system.

#### Anterior view of a biting behavior

To visualize the axial view of the animal during feeding behaviors and to show the different configurations of the radula (opened and closed), biting behaviors were elicited and video recorded in an unconstrained animal (Figure S1). The animal was placed in a behavioral aquarium filled with artificial seawater (16°C). A webcam (Logitech C920, 30 FPS) was mounted on a tripod to provide a top-down view of the behavioral arena. The animal was enticed to the surface of the water with pieces of dried nori. Biting behavior was then elicited by stimulating the lips and anterior tentacles of the animal with pieces of nori attached to wooden dowels. One biting sequence in which the animal faced the camera throughout the behavior was chosen for visualization.

#### Biomechanical model of the buccal mass

##### Variable convention

Throughout this document, scalar values are denoted with italicized letters (Latin or Greek), vectors are stylized with an arrow ($\vec{x}$), unit vectors are stylized with a hat ($\hat{t}$), and matrices are indicated with bold typeface (***J***).

##### Mechanics of a beam structure

The “hinge” is the anatomical structure in the buccal mass that connects the odontophore to the I1/I3 complex and is formed by the interdigitation of the I1/I3, I2, I4, and I6 muscles.^15^ This interdigitation spans much of the ventral half of the odontophore mediolaterally, and a thick band of tissue runs from the I6 muscle, through the hinge, to the ventral I3.^15^ The anatomy of the hinge structure, with its thick midsagittal band and extensive mediolateral components, suggests that this muscle may passively generate forces not only by being stretched but also by being bent or folded. To capture this combined axial and bending stiffness, here we model the hinge muscle as a geometrically exact beam (GEB)^34^^,^^67^ (Figure S10A). A GEB is a 1-dimensional continuum element that can resist both forces and moments and is a geometrically nonlinear extension of classical Euler-Bernoulli Beam Theory. The configuration of the beam is captured by its planar position ((*x*, *y*)) and its heading angle (*θ*) at each point along the beam (Figure S10B). In classical formulations and those followed here, a linear constitutive model is assumed, such that axial force and bending moments are linearly proportional to axial strain and curvature, respectively.^34^^,^^67^ We utilize that assumption here, attributing any observed nonlinearity in the mechanical data to geometric nonlinearities. The governing equations were modified from Voesenek et al.^34^ to convert their dynamical equations into quasistatic ones. A quasistatic formulation was used because previous work has shown that *Aplysia* feeding is quasistatic throughout maturation.^7^^,^^33^ We point readers to Voesenek et al.^34^ for a complete derivation of the governing equations.

Briefly, the quasistatic governing equations for the continuum beam element are derived from the instantaneous force and moment balance on each infinitesimal segment of the beam (Figure S10C). In the absence of body forces (e.g., gravity), this results in the following set of coupled partial differential equations (PDEs).

(Equation 1) $$\begin{array}{c} \frac{\partial }{\partial s}N\left(s\right)\hat{t}\left(s\right)+\frac{\partial }{\partial s}Q\left(s\right)\hat{n}\left(s\right)=0 \end{array}$$

(Equation 2) $$\begin{array}{c} \frac{\partial }{\partial s}M\left(s\right)+Q\left(s\right)=0 \end{array}$$

where *s* is the position along the arc length of the beam, *N*(*s*) and *Q*(*s*) are the axial and shear forces at the point *s*, $\hat{t}\left(s\right)$ and $\hat{n}\left(s\right)$ are the tangent and normal vectors of the beam, and *M*(*s*) is the bending moment. These are coupled with constitutive equations

(Equation 3) $$\begin{array}{c} N\left(s\right)=\mu_{\epsilon }\epsilon \left(s\right) \end{array}$$

(Equation 4) $$\begin{array}{c} M\left(s\right)=\eta_{\kappa }\left(\kappa \left(s\right)-\kappa_{0}\left(s\right)\right) \end{array}$$

where *μ*_*ϵ*_ and *η*_*κ*_ are the axial stiffness (units mN) and bending stiffness (units mN·mm^2^), *ϵ*(*s*) is the axial strain (unitless), and *κ*(*s*) is the curvature in the beam (units mm^−1^). *κ*_0_(*s*) is the curvature of the beam in the rest configuration. The axial strain is calculated from the position of the beam’s centerline (*x*(*s*), *y*(*s*)) (Figure S10B) as

(Equation 5) $$\begin{array}{c} \epsilon \left(s\right)=\sqrt{{\left(\frac{\partial x}{\partial s}\right)}^{2}+{\left(\frac{\partial y}{\partial s}\right)}^{2}}-1 \end{array}$$

and the curvature is calculated from the heading angle *θ*(*s*) as

(Equation 6) $$\begin{array}{c} \kappa \left(s\right)=\frac{\partial \theta }{\partial s} \end{array}$$

Finally, the heading angle is calculated from the centerline positions as

(Equation 7) $$\begin{array}{c} \theta \left(s\right)=\mathrm{atan}\left(\frac{\partial y/\partial s}{\partial x/\partial s}\right) \end{array}$$

and the tangent and normal vectors can be found using the heading angle as

(Equation 8) $$\begin{array}{c} \hat{t}\left(s\right)=\left[\begin{array}{c} \cos\;\theta \left(s\right)\\ \sin\;\theta \left(s\right) \end{array}\right] \end{array}$$

and

(Equation 9) $$\begin{array}{c} \hat{n}\left(s\right)=\left[\begin{array}{c} -\sin\;\theta \left(s\right)\\ \cos\;\theta \left(s\right) \end{array}\right] \end{array}$$

This is a coupled first-order system with the state vector $\vec{X}=\left[x\left(s\right),y\left(s\right),\theta \left(s\right),N\left(s\right),Q\left(s\right),M\left(s\right)\right]$. This system requires six boundary conditions to be properly constrained.

##### Hybrid kinetic/kinematic model of the buccal mass

To investigate the effect of buccal mass reconfiguration on grasper protraction in *Aplysia* biting and rejection behaviors, we developed a hybrid kinetic/kinematic model of the buccal mass, where user-specified kinematic degrees of freedom interact with a biomechanical model of the surrounding tissue (Figure S11). For the work presented here, we assume the buccal mass is bilaterally symmetrical, such that only structures in the midsagittal plane need to be directly modeled, and effects from other structures can be projected onto the midsagittal plane. The buccal mass model consists of a rigid ellipse, modeling the shape of the odontophore, connected to a geometrically exact beam, modeling the hinge muscle structure (Figure S11A). The tubelike I3 muscle, in the midsagittal plane has a dorsal and ventral element, which we approximate using two rigid bars. The hinge beam connects ventrally to the rigid bar approximating the rest geometry of the ventral I1/I3 complex and dorsally to a rigid bar representing the I6 muscle. The I6 muscle is internal to the odontophore, and, in the model, serves as the connection point of the hinge to the odontophore. Finally, the I2 model is approximated as a chord that attaches to the posterior edges of the dorsal and ventral I1/I3 bars (anatomically called the lateral groove) and wraps conformally around the odontophore.

The forces and torques produced by each model element are used to determine the overall loading on the odontophore ellipse. The tension in the I2 muscle is modeled as a linear combination of a passive tension that is linearly proportional to the stretch *λ*_*I*2_ ($\lambda_{I2}=L_{I2}\;/\;L_{I2}^{0}$, where *L*_*I*2_ and $L_{I2}^{0}$ are the current and rest lengths of the I2, respectively) and an active tension. This active tension is modeled using a simplified version of the Hill muscle model presented in Yu et al.^13^ Here,

(Equation10) $$\begin{array}{c} T_{I2}^{act}\left(A_{I2},\lambda_{I2}\right)=T_{I2}^{\max}A_{I2}FL_{I2}\left(\lambda_{I2}\right) \end{array}$$

where $T_{I2}^{\max}=240\;mN$ is the maximum isometric force the I2 can generate,^14^ and *A*_*I*2_ ∈ [0,1] is the normalized activation of I2. As the dynamics of the system are not considered in this analysis, this activation is simply a control variable that is set during computational experiments. *FL*(*λ*_*I*2_) is the length-tension relationship of the I2 muscle from Yu et al.^13^:

(Equation 11) $$\begin{array}{c} FL\left(\lambda_{I2}\right)=1.8-10.8\;\lambda_{I2}+19.2\;\lambda_{I2}^{2}-9.2\;\lambda_{I2}^{3} \end{array}$$

Note that the constant term has been modified from the original paper (originally 1.81) so that the length-tension curve equals 1 at *λ*_*I*2_ = 1. Additionally, in the original Yu model, the length-tension curve was a function of length normalized by the optimal muscle length.^13^ Here, we assume the optimal muscle length occurs at its rest length. The total tension I2 is then

(Equation 12) $$\begin{array}{c} T_{I2}\left(A_{I2},\lambda_{I2}\right)=T_{I2}^{act}\left(A_{I2},\lambda_{I2}\right)+k_{I2}\left(\lambda_{I2}-1\right) \end{array}$$

where *k*_*I*2_ = 800 mN is the passive stiffness of the I2 muscle. This value of *k*_*I*2_ was chosen such that the passive force reached $T_{I2}^{\max}$ at a stretch of 1.3 as observed in the animal tissue.^13^ To translate this I2 tension into its corresponding force vector and torque, we calculate the I2’s mechanical advantage. For a small arc of the I2 where it is in contact with the odontophore (Figure S11B), the force from the odontophore is balanced by the tension in the I2, which acts tangent to the muscle on each side of the arc. Therefore, the force vector from the odontophore on this arc is equal to the difference in the tangent vectors across the arc. For an infinitesimal arc spanning the elliptical parameter interval *dϕ*, the net force vector on the odontophore, $d{\vec{F}}_{I2}$, is

(Equation 13) $$\begin{array}{c} d{\vec{F}}_{I2}=T_{I2}\left(A_{I2},\lambda_{I2}\right)\frac{\partial \hat{t}}{\partial \phi } \end{array}$$

Here, *ϕ* ∈ [0,2*π*] is the parameter of the ellipse boundary, such that for a point (*x*, *y*) on the boundary of a standard ellipse is found as *x* = *a cos ϕ* and *y* = *b sin ϕ*. The total force vector that I2 applies to the odontophore is then the integral of $d{\vec{F}}_{I2}$ along the arclength of the boundary where I2 is in contact. This simplifies to

(Equation 14) $$\begin{array}{c} {\vec{F}}_{I2}=T_{I2}\left(A_{I2},\lambda_{I2}\right)\left({\hat{t}}_{1}+{\hat{t}}_{2}\right) \end{array}$$

(Figure S11B). The moment generated by the I2 is

(Equation 15) $$\begin{array}{c} M_{I2}=T_{I2}\left(A_{I2},\lambda_{I2}\right)\int_{\phi_{1}}^{\phi_{2}}{\vec{r}}_{I2}\times \frac{\partial \hat{t}}{\partial \phi }\;d\phi \end{array}$$

where ${\vec{r}}_{I2}$ (the moment arm of the arc of I2) is the vector pointing from the hinge attachment point on the odontophore to the point on the odontophore boundary, and *ϕ*_1_ and *ϕ*_2_ are the ellipse parameters where the I2 starts and stops being in contact with the odontophore. Here, × denotes the 2D cross product, from which only the scalar magnitude is considered as the model is planar. This value is integrated numerically (*trapz*, MATLAB 2024a, MathWorks).

The beam hinge model produces passive forces and torques following the constitutive equations outlined above. For the simulations performed here, the active contributions of the hinge were not considered as the hinge is most strongly activated during the retraction phase of behaviors,^15^ and we are focused on protraction-phase configurations of the buccal mass. The beam is parameterized such that the *s* = 0 end connects to the ventral lateral groove, and the *s* = *L* end (where *L* is the arclength of the beam) is connected to the odontophore at the I6 muscle. The boundary conditions for the beam equations are specified using the other components of the buccal mass model. At the *s* = 0 end, the kinematic variables ([*x*(*s*), *y*(*s*), *θ*(*s*)]) are fixed to be equal to their initial conditions (no displacement). At the *s* = *L* end, the kinetic variables ([*N*(*s*) *Q*(*s*), *M*(*s*)]) are specified based on the net forces and moments (about the point where the odontophore connects to the hinge) applied by the other elements of the system to the odontophore. Specifically,

(Equation 16) $$\begin{array}{c} \left[\begin{array}{c} N\left(s=L\right)\\ Q\left(s=L\right) \end{array}\right]=R{\left(\theta \;\left(s=L\right)\right)}^{T}\vec{F} \end{array}$$

where ***R***(*θ*) is the 2D rotation matrix and $\vec{F}$ is the total force vector acting on the odontophore.

##### Kinematic constraints and contact forces

To investigate the role that shape changes in the odontophore and the I1/I3 lumen have on protraction, we apply additional kinematic constraints on the system. These constraints are controlled during computational experiments to set different configurations of the system (See Computational Experiments).

The first kinematic constraint relates to the shape of the odontophore and how it attaches to the hinge beam (Figure S11C). Here, we adopt the odontophore shape change model utilized in Novakovic et al.^16^ in which the aspect ratio of the odontophore ellipse (Φ = major axis/minor axis) is the free parameter. The aspect ratio takes values between 1.1 (fully open odontophore) and 1.8 (fully closed odontophore). To determine the lengths of the two axes for a given aspect ratio, we use the same assumptions from Novakovic et al.^16^ that the odontophore is an isovolumetric ellipsoid and that the mediolateral radius is the same as the minor axis in the midsagittal plane. Let the major axis be *R*_1_ and the minor axis be *R*_2_. The volume of the ellipsoid is then

(Equation 17) $$\begin{array}{c} V=\frac{4}{3}\pi R_{1}R_{2}^{2} \end{array}$$

and the aspect ratio is Φ = *R*_1_/*R*_2_. Then, for a specified aspect ratio, we can calculate the major and minor axes as

(Equation 18) $$\begin{array}{c} R_{2}={\left(\frac{3V}{4\pi \Phi }\right)}^{\frac{1}{3}} \end{array}$$

(Equation 19) $$\begin{array}{c} R_{1}=\Phi R_{2} \end{array}$$

The value for the fixed volume is calculated using *R*_1_ and *R*_2_ in the rest configuration. The location ${\left([x_{g},y_{g}\right]}^{T})$ and orientation (*θ*_*g*_) of the ellipse are then determined by the location (${\left[x_{H},y_{H}\right]}^{T}$) and orientation (*θ*_*I*6_) of the *s* = *L* end of the beam hinge (Figure S11C). We assume that the location of the connection between the beam and the ellipse occurs (1) at the same polar angle from the long axis of the ellipse (*θ*_*H*_: constant) and (2) at a fixed distance from the edge of the ellipse in that same polar direction (Δ*R*: constant) (Figure S11C). Finally, (3) the angle between the I6 bar and the vector from the hinge point and the ellipse center is fixed (*ϕ*_*H*_: constant). These three constraints fully defined the position and orientation of the ellipse for a given configuration of the beam. The orientation of the grasper ellipse (*θ*_*g*_) is found as

(Equation 20) $$\begin{array}{c} \theta_{g}=\theta_{I6}+\phi_{H}-\left(\theta_{H}-\pi \right) \end{array}$$

The position of the grasper ellipse is

(Equation 21) $$\begin{array}{c} \left[\begin{array}{c} x_{g}\\ y_{g} \end{array}\right]=\left[\begin{array}{c} x_{H}\\ y_{H} \end{array}\right]+R_{H}\left[\begin{array}{c} \cos\left(\theta_{I6}+\phi_{H}\right)\\ \sin\left(\theta_{I6}+\phi_{H}\right) \end{array}\right] \end{array}$$

where *R*_*H*_ is the distance from the center of the ellipse to the *s* = *L* tip of the beam:

(Equation 22) $$\begin{array}{c} R_{H}=\frac{R_{1}R_{2}}{\sqrt{{\left(R_{1}\;\sin\left(\theta_{H}\right)\right)}^{2}+{\left(R_{2}\;\cos\left(\theta_{H}\right)\right)}^{2}}}-\Delta R \end{array}$$

Here, the first term is the effective radius of the ellipse at the polar angle *θ*_*H*_ and will change based on the aspect ratio of the ellipse (through the values of *R*_1_ and *R*_2_). Because this constraint deals only with equality constraints, it is satisfied exactly.

The second kinematic constraint deals with the shape of the I3 lumen and how the I3 contacts the odontophore (Figure S11D). In the animal, the lateral groove shortens at the peak of protraction during biting.^31^ To approximate the effects of this in the model, we posteriorly depress the dorsal line of the I1/I3 complex and simulate the consequent contact forces on the odontophore. Only the dorsal lateral groove point (${\left[x_{d},y_{d}\right]}^{T}$) is changed by this constraint; the jaw location and the ventral I1/I3 line remain fixed. This constraint is applied by specifying the stretch in the lateral groove (*λ*_*LG*_) and calculating the requisite depression angle of the dorsal I1/I3 line relative to the horizontal (Figure S11D). Specifically, the new dorsal lateral groove point is found as

(Equation 23) $$\begin{array}{c} \left[\begin{array}{c} x_{d}\\ y_{d} \end{array}\right]=\left[\begin{array}{c} x_{j,d}\\ y_{j,d} \end{array}\right]+R_{d}\left[\begin{array}{c} \cos\;\theta_{d}\left(\lambda_{\mathrm{LG}}\right)\\ \sin\;\theta_{d}\left(\lambda_{\mathrm{LG}}\right) \end{array}\right] \end{array}$$

where

(Equation 24) $$\begin{array}{c} \theta_{d}\left(\lambda_{\mathrm{LG}}\right)=\frac{3\pi }{2}-\left(\gamma \left(\lambda_{\mathrm{LG}}\right)+\beta \right) \end{array}$$

is the angle from the horizontal to the dorsal lateral I3 line, $\beta =\mathrm{asin}\frac{R_{v,x}}{L_{1}}$, *R*_*v*,*x*_ is the x projection of the ventral I3 line, and *L*_1_ is the diagonal length of the I3 lumen. Finally,

(Equation 25) $$\begin{array}{c} \gamma \left(\lambda_{\mathrm{LG}}\right)=\mathrm{acos}\frac{L_{1}^{2}+R_{d}^{2}-{\left(\lambda_{\mathrm{LG}}L_{\mathrm{LG}\;0}\right)}^{2}}{2L_{1}R_{d}} \end{array}$$

where *L*_*LG*0_ is the rest length of the lateral groove. The effects of this constraint on the odontophore are captured through a contact force (${\vec{F}}_{con}$, Figure S11D). In the animal, this contact may not predominantly occur in the midsagittal plane but will be distributed in 3D across the top surface of the odontophore. To approximate this, we assume the contact force is applied to the average position of the ellipse boundary points that are in the top half of the I1/I3 lumen

(Equation 26) $$\begin{array}{c} \left[\begin{array}{c} x_{con}\\ y_{con} \end{array}\right]=\frac{1}{N}\sum_{i}^{N}\left[\begin{array}{c} x_{ellipse,i}\\ y_{ellipse,i} \end{array}\right]\delta_{i} \end{array}$$

where

(Equation 27) $$\begin{array}{c} \left[\begin{array}{c} x_{ellipse,i}\\ y_{ellipse,i} \end{array}\right]=\left[\begin{array}{c} x_{g}\\ y_{g} \end{array}\right]+R\left(\theta_{g}\right)\left[\begin{array}{c} R_{1}\;\cos\;\phi_{i}\\ R_{2}\;\sin\;\phi_{i} \end{array}\right] \end{array}$$

are the boundary points of the grasper ellipse, *N* is the number of points along the grasper ellipse boundary, and

(Equation 28) $$\begin{array}{c} \delta_{i}= \end{array}\left\{\begin{array}{ll} 1 & x_{ellipse,i}>\left[\frac{x_{v}+x_{d}}{2}\right]\;\text{and}\;y_{ellipse,i}>\left[\frac{y_{j,d}+y_{j,v}}{2}\right]\\ 0 & \mathrm{else} \end{array}\right)$$

The magnitude of the contact force is linearly proportional to the perpendicular distance from ${\left[x_{con},y_{con}\right]}^{T}$ to a line parallel to and a fixed distance from the dorsal I3 line (white dashed line in Figure S11D). The contact force always points perpendicularly to the dorsal I3 line.

Finally, to prevent the odontophore from passing through the ventral I1/I3 and to approximate the contact with the ventral I1/I3 complex, a force is applied to any point on the boundary of the ellipse that penetrates the ventral I1/I3 line. The magnitude of the force is linearly proportional to the perpendicular distance of the point to the ventral I1/I3 line. The total force (moment) from this constraint was found by integrating the force (moment) at each point along the boundary.

All constants associated with the kinematic constraints were estimated from the model’s resting configuration using the inverted forms of (Equation 17), (Equation 18), (Equation 19), (Equation 20), (Equation 21), (Equation 22), (Equation 23), (Equation 24), (Equation 25), 18, 19, 20, 21, 22, 23, 24, and 25.

##### Implementation of the mechanical model

The governing PDEs of the model were discretized using a finite difference approximation (fourth order approximation for central points, third order approximation for first (last) two boundary points^68^), and the resulting system of equations was simultaneously solved using a custom adaptive implementation of Broyden’s method.^69^ Briefly, Broyden’s method is a quasi-Newton method approach to solving nonlinear equations of the form $\vec{f}\left(\vec{X}\right)=0$, where $\vec{f}\left(\vec{X}\right)$ is a system of nonlinear functions of the vector $\vec{X}$. The solution to this system of equations is approximated iteratively using the update rule:

(Equation 29) $$\begin{array}{c} {\vec{X}}_{i+1}={\vec{X}}_{i}-\alpha J_{i}^{-1}\vec{f}\left(\vec{X}\right)={\vec{X}}_{i}+\Delta {\vec{X}}_{i} \end{array}$$

where $J=\partial \vec{f}\;/\partial \vec{X}$ is the Jacobian of the system, and *α* ∈ [0,1] is a scaling factor. Broyden’s method is a quasi-Newton method because, instead of recomputing and inverting the Jacobian of the system at each step, the inverse of the Jacobian is calculated once at the initial condition of the solver and then updated using a rank-1 update based on the step size and change in the function value:

(Equation 30) $$\begin{array}{c} J_{i+1}^{-1}=J_{i}^{-1}+\frac{1}{\Delta {\vec{X}}_{i}^{T}J_{i}^{-1}\Delta {\vec{f}}_{i}\;}\left(\Delta {\vec{X}}_{i}-J_{i}^{-1}\Delta {\vec{f}}_{i}\right)\left(\Delta {\vec{X}}_{i}^{T}\right)J_{i}^{-1} \end{array}$$

where $\Delta {\vec{f}}_{i}=\vec{f}\left({\vec{X}}_{i}\right)-\vec{f}\left({\vec{X}}_{i-1}\right)$. In the solver used here, the initial Jacobian of the system was also calculated using a finite difference approximation. This solver was made adaptive in two ways. First, within a given timestep of the simulation, to minimize the likelihood of oscillations in the solver, the value of *α* was decreased for each iteration as

(Equation 31) $$\begin{array}{c} \alpha_{i}={\left(1-\frac{i}{i_{\max}+1}\right)}^{3} \end{array}$$

where *i*_*max*_ = 100 is the maximum allowed number of iterations. Second, for a given timestep, if the solver failed to find a solution within the allowed 100 iterations, then the timestep was subdivided into smaller steps, where the number of subdivisions *N*_*sub*_ was iteratively updated as

(Equation 32) $$\begin{array}{c} N_{sub,j+1}=\lfloor 1.1\times \left(N_{sub,j}+1\right)\rfloor \end{array}$$

where ⌊*x*⌋ is the floor function.

To avoid numerical instabilities caused by large differences in the order of magnitude of values in the state vector (e.g., between displacements and forces), we adopt the same equation scaling procedure laid out in Voesenek et al.^34^ for the PDEs. Based on a convergence analysis, 30 nodes were used to discretize the beam. At this level of resolution, peak force predictions in calibration experiments differed from a reference simulation with 100 nodes by less than 0.1%. All code was implemented using custom MATLAB code (R2024a, MathWorks).

#### Parameter estimation

##### Anatomical parameter estimation

The rest geometry of the biomechanical model was approximated using points and structures digitized from the midsagittal anatomical image presented in Neustadter et al.^31^ using WebPlotDigitizer^64^ (Figure S12A). Specifically, these structures included 1) the boundary profile of the odontophore (magenta squares); 2) the dorsal edge of the I6 (black up-pointing triangle); 3) the anterior edge of the ventral I3 (blue down-pointing triangle); 4) the anterior and posterior edges of the dorsal I3 (blue right-pointing triangles); and 5) a midline curve connecting the I6 through the hinge to the ventral I3 (green circles). The boundary profile points were used to fit the ellipse center and radii (Figure S11C). To allow the hinge beam to be discretized with any required resolution for the finite difference solver, the midline curve points were fit to a cubic Bezier spline curve that could then be resampled. We parameterized the beam such that *s* = 0 is the ventral edge of the beam connected to the ventral I3 rod, and *s* = *L* is the dorsal edge of the beam connected to the I6 rod.

##### Scale reconciliation

The data used to fit the anatomical and mechanical parameters (See “Constitutive model parameter calibration”) were obtained from animals of different sizes. To account for potential size-dependent effects, we rescaled the anatomy of the model to match the approximate size of the animals used in the mechanical studies presented in Sutton et al.^15^ We chose to scale the anatomy and not the force-displacement data because we do not know *a priori* how the force properties of a beam structure will scale with mass, but Rogers et al.^33^ recently showed that the midsagittal anatomy of the buccal mass scales isometrically. Data from Rogers et al.^33^ were digitized (Figure S12B) using WebPlotDigitizer^64^ to obtain the appropriate scaling relationships, and the scaling exponents were set for exact isometry (length ∝ mass^1/3^).

To perform this rescaling, the buccal mass masses used in each dataset were estimated, as the animal masses for the individual animals were not reported. From the Neustadter et al.^31^ anatomy, we measured both the dorsoventral height and anteroposterior length of the buccal mass according to the measurements reported in Rogers et al.^33^ (Figure S12C). Using the scaling relationships from Rogers et al.,^33^ we obtained two estimates of the mass of the buccal mass (from height: 4.1 g; from length: 3.2 g), and the mass of the animal’s buccal mass was estimated as the geometric mean of these two values (3.6 g, corresponding to a body mass of 455 g). The geometric mean was used because this would provide the middle point on the logarithmic scale of masses used in the scaling analysis. Then, from the Sutton et al.^15^ force-displacement data (See “Constitutive model parameter calibration”), the buccal mass anteroposterior length was obtained from the displacement data, which were reported in both centimeters and buccal mass lengths. Again, using the Rogers et al.^33^ scaling laws, the mass of the buccal mass of this animal was estimated as 2.3 g (body mass: 285 g). All length measurements in the anatomical model were then rescaled by (2.3/3.6)^1/3^ to obtain the approximate anatomy of the 285 g animal used by Sutton et al*.*^15^ This rescaled anatomy was used for all calibration and computational experiments.

##### Constitutive model parameter calibration

To calibrate the stiffness parameters of the beam model (*μ*_*ϵ*_ and *η*_*κ*_), we simulated the experiments presented by Sutton et al.^15^ Briefly, in those experiments, the buccal mass was excised, and the I3 lumen was dorsally bisected to expose the odontophore. The odontophore was left connected only by the hinge, and the I3 tissue was pinned out. Then sutures were attached between the odontophore and a force transducer. The transducer was displaced in fixed increments, and the reaction force generated by the hinge was recorded. Displacements were reindexed to the level just after the force was first registered on the force transducer. Data were digitized for the representative animal reported in Sutton et al. Figure 5^15^ using WebPlotDigitizer.^64^

In our simulated experiments, we attached a stiff simulated spring to the dorsal edge of the I6 bar and applied displacements to the other edge of the spring in the direction of the ventral I1 bar. We calculated the displacement of the odontophore as the projection of the displacement of the tip of the ellipse along the direction of the ventral I1 bar. The reaction force was calculated using the stretch of the simulated spring. This allows for the calculation of the analogous force-displacement curve to compare with the Sutton data.^15^ The mechanical parameters *μ*_*ϵ*_ and *η*_*κ*_ were optimized to minimize the squared error with the animal data within behaviorally relevant displacements. The squared error was minimized numerically using the MATLAB function *fminsearch* (R2024a, MathWorks).

#### Computational experiments

To investigate the effects of the odontophore and I1/I3 shape change on the protraction of the odontophore, we conducted the following computational experiment using the presented biomechanical model of the buccal mass. Note that the time series of odontophore and lateral groove shape change followed in these experiments do not correspond to a specific feeding behavior but rather allow us to efficiently cover the full kinematic parameter space. As the mechanics of the model and animal system are quasistatic,^33^ and therefore, not history-dependent, the configurations of the buccal mass obtained in these computational experiments are still relevant to behaviors even if the path between configurations is different. In these experiments, the odontophore is protracted by activating the I2 while the aspect ratio of the odontophore is changed from its rest shape to a specified final aspect ratio. Once the I2 has reached the specified maximum value of activation and the aspect ratio has reached its final value, the length of the lateral groove is changed from 1.1*L*_*LG*0_ to 0.72*L*_*LG*0_, where *L*_*LG*0_ is the rest length of the lateral groove. These extremes were chosen based on *in vivo* kinematics measurements from biting and rejection to cover the *in vivo* kinematic parameter range. Throughout the experiment, the horizontal distance between the jaw line and the anterior-most point on the odontophore (referred to as the translation of the odontophore, Δ*x*) and the angle between the I6 and the jaw line (called the rotation of the odontophore, *θ*_*I*6_) were recorded as analogous measurements to those reported in Neustadter et al.^31^ and those measured during the kinematic analysis in this study. Data were collected during the shortening of the lateral groove for multiple simulations with different final aspect ratios. From these data, we could cover the full behaviorally relevant configuration space of aspect ratio and lateral groove stretch. The 2D translation and rotation data were then interpolated using a local quadratic regression (using the MATLAB *fit* function for a ‘loess’ fit type, span = 0.3, normalize = ‘on’, MATLAB 2024a, MathWorks) to create contour plots of translation and rotation over the configuration space (Figures S6 and S7). These same interpolated functions were used to create the contour cross-sections reported in the main manuscript (Figures 3C and 3D). This process was repeated for maximum I2 activations of 15%, 40%, 65%, and 90%.

### Quantification and statistical analysis

The comparisons of model predictions with animal data were based on single representative behaviors. Therefore, there are no quantification or statistical analyses to include in this study.

## References

[1] Kier W.M., Smith K.K. (1985). Tongues, tentacles and trunks: the biomechanics of movement in muscular-hydrostats. Zool. J. Linn. Soc..

[2] Schulz A.K., Schneider N., Zhang M., Singal K. (2023). A Year at the Forefront of Hydrostat Motion. Biol. Open.

[3] Valero-Cuevas F.J., Santello M. (2017). On neuromechanical approaches for the study of biological and robotic grasp and manipulation. J. NeuroEng. Rehabil..

[4] Moulton D.E., Lessinnes T., O’Keeffe S., Dorfmann L., Goriely A. (2016). The elastic secrets of the chameleon tongue. Proc. R. Soc. A A..

[5] Ellers O., Ellers K.-I., Johnson A.S., Po T., Heydari S., Kanso E., McHenry M.J. (2024). Soft skeletons transmit force with variable gearing. J. Exp. Biol..

[6] Uyeno T.A., Clark A.J. (2015). Muscle Articulations: Flexible Jaw Joints Made of Soft Tissues. Integr. Comp. Biol..

[7] Bennington M.J., Liao A.S., Sukhnandan R., Kundu B., Rogers S.M., Gill J.P., McManus J.M., Sutton G.P., Chiel H.J., Webster-Wood V.A. (2025). Incorporating buccal mass planar mechanics and anatomical features improves neuromechanical modeling of *Aplysia* feeding behavior. Biol. Cybern..

[8] Cropper E.C., Jing J., Weiss K.R., Byrne J.H. (2017). The Oxford Handbook of Invertebrate Neurobiology.

[9] Gill J.P., Chiel H.J. (2020). Rapid adaptation to changing mechanical load by ordered recruitment of identified motor neurons. eNeuro.

[10] Hurwitz I., Susswein A.J. (1996). B64, a newly identified central pattern generator element producing a phase switch from protraction to retraction in buccal motor programs of *Aplysia californica*. J. Neurophysiol..

[11] Tam S., Hurwitz I., Chiel H.J., Susswein A.J. (2020). Multiple local synaptic modifications at specific sensorimotor connections after learning are associated with behavioral adaptations that are components of a global response change. J. Neurosci..

[12] Whim M.D., Lloyd P.E. (1990). Neuropeptide cotransmitters released from an identified cholinergic motor neuron modulate neuromuscular efficacy in *Aplysia*. J. Neurosci..

[13] Yu S.N., Crago P.E., Chiel H.J. (1999). Biomechanical properties and a kinetic simulation model of the smooth muscle I2 in the buccal mass of *Aplysia*. Biol. Cybern..

[14] Sutton G.P., Mangan E.V., Neustadter D.M., Beer R.D., Crago P.E., Chiel H.J. (2004). Neural control exploits changing mechanical advantage and context dependence to generate different feeding responses in *Aplysia*. Biol. Cybern..

[15] Sutton G.P., Macknin J.B., Gartman S.S., Sunny G.P., Beer R.D., Crago P.E., Neustadter D.M., Chiel H.J. (2004). Passive hinge forces in the feeding apparatus of *Aplysia* aid retraction during biting but not during swallowing. J. Comp. Physiol. A Neuroethol. Sens. Neural Behav. Physiol..

[16] Novakovic V.A., Sutton G.P., Neustadter D.M., Beer R.D., Chiel H.J. (2006). Mechanical reconfiguration mediates swallowing and rejection in *Aplysia californica*. J. Comp. Physiol. A Neuroethol. Sens. Neural Behav. Physiol..

[17] Webster-Wood V.A., Gill J.P., Thomas P.J., Chiel H.J. (2020). Control for multifunctionality : bioinspired control based on feeding in *Aplysia californica*. Biol. Cybern..

[18] Sukhnandan R., Chen Q., Shen J., Pao S., Huan Y., Sutton G.P., Gill J.P., Chiel H.J., Webster-Wood V.A. (2024). Full Hill-type muscle model of the I1/I3 retractor muscle complex in *Aplysia californica*. Biol. Cybern..

[19] Ye H., Morton D.W., Chiel H.J. (2006). Neuromechanics of multifunctionality during rejection in *Aplysia californica*. J. Neurosci..

[20] Li Y., Webster-Wood V.A., Gill J.P., Sutton G.P., Chiel H.J., Quinn R.D. (2024). A computational neural model that incorporates both intrinsic dynamics and sensory feedback in the *Aplysia* feeding network. Biol. Cybern..

[21] Cash D., Carew T.J. (1989). A quantitative analysis of the development of the central nervous system in juvenile *Aplysia californica*. J. Neurobiol..

[22] Lu H., McManus J.M., Chiel H.J. (2013). Extracellularly Identifying Motor Neurons for a Muscle Motor Pool in *Aplysia californica*. JoVE J..

[23] Howells H. (1942). The Structure and Function of the Alimentary Canal of *Aplysia punctata*. J. Cell Sci..

[24] Kehl C.E., Wu J., Lu S., Neustadter D.M., Drushel R.F., Smoldt R.K., Chiel H.J. (2019). Soft-surface grasping: Radular opening in *Aplysia californica*. J. Exp. Biol..

[25] Evans C.G., Rosen S., Kupfermann I., Weiss K.R., Cropper E.C. (1996). Characterization of a radula opener neuromuscular system in *Aplysia*. J. Neurophysiol..

[26] Morton D.W., Chiel H.J. (1993). The timing of activity in motor neurons that produce radula movements distinguishes ingestion from rejection in *Aplysia*. J. Comp. Physiol..

[27] Lu H., Mcmanus J.M., Cullins M.J., Chiel H.J. (2015). Preparing the periphery for a subsequent behavior: Motor neuronal activity during biting generates little force but prepares a retractor muscle to generate larger forces during swallowing in *Aplysia*. J. Neurosci..

[28] McManus J.M., Lu H., Cullins M.J., Chiel H.J. (2014). Differential activation of an identified motor neuron and neuromodulation provide *Aplysia*’s retractor muscle an additional function. J. Neurophysiol..

[29] Hurwitz I., Neustadter D., Morton D.W., Chiel H.J., Susswein A.J. (1996). Activity patterns of the B31/B32 pattern initiators innervating the I2 muscle of the buccal mass during normal feeding movements in *Aplysia californica*. J. Neurophysiol..

[30] Kupfermann I. (1974). Feeding behavior in *Aplysia*: a simple system for the study of motivation. Behav. Biol..

[31] Neustadter D.M., Herman R.L., Drushel R.F., Chestek D.W., Chiel H.J. (2007). The kinematics of multifunctionality: Comparisons of biting and swallowing in *Aplysia californica*. J. Exp. Biol..

[32] Schindelin J., Arganda-Carreras I., Frise E., Kaynig V., Longair M., Pietzsch T., Preibisch S., Rueden C., Saalfeld S., Schmid B. (2012). Fiji: An open-source platform for biological-image analysis. Nat. Methods.

[33] Rogers S.M., Gill J.P., De Campos A.S., Wang K., Kaza I.V., Fan V.X., Sutton G.P., Chiel H.J. (2024). Scaling of buccal mass growth and muscle activation determine the duration of feeding behaviors in the marine mollusc *Aplysia californica*. J. Exp. Biol..

[34] Voesenek C.J., Li G., Muijres F.T., van Leeuwen J.L. (2020). Experimental-numerical method for calculating bending moments in swimming fish shows that fish larvae control undulatory swimming with simple actuation. PLoS Biol..

[35] Cullins M.J., Gill J.P., Mcmanus J.M., Lu H., Shaw K.M., Chiel H.J. (2015). Sensory Feedback Reduces Individuality by Increasing Variability within Subjects. Curr. Biol..

[36] Dagenais P., Hensman S., Haechler V., Milinkovitch M.C. (2021). Elephants evolved strategies reducing the biomechanical complexity of their trunk. Curr. Biol..

[37] Flash T., Zullo L. (2023). Biomechanics, motor control and dynamic models of the soft limbs of the octopus and other cephalopods. J. Exp. Biol..

[38] Ross C.F., Laurence-Chasen J.D., Li P., Orsbon C., Hatsopoulos N.G. (2024). Biomechanical and Cortical Control of Tongue Movements During Chewing and Swallowing. Dysphagia.

[39] Chiel H.J., Beer R.D. (1997). The brain has a body: Adaptive behavior emerges from interactions of nervous system, body and environment. Trends Neurosci..

[40] Ijspeert A.J., Daley M.A. (2023). Integration of feedforward and feedback control in the neuromechanics of vertebrate locomotion: a review of experimental, simulation and robotic studies. J. Exp. Biol..

[41] Full R.J., Koditschek D.E. (1999). Templates and anchors: Neuromechanical hypotheses of legged locomotion on land. J. Exp. Biol..

[42] Moore J.R., Vigoreaux J.O. (2007). *Nature’s Versatile Engine: Insect Flight Muscle Inside and Out* 44–60.

[43] Dickinson M. (2006). Insect flight. Curr. Biol..

[44] Campbell K.B., Chandra M. (2006). Functions of stretch activation in heart muscle. J. Gen. Physiol..

[45] Pfeifer R., Iida F., Lungarella M. (2014). Cognition from the bottom up: On biological inspiration, body morphology, and soft materials. Trends Cogn. Sci..

[46] Rose R.M., Benjamin P.R. (1979). The relationship of the central motor pattern to the feeding cycle of *Lymnaea stagnalis*. J. Exp. Biol..

[47] Uyeno T.A., Kier W.M. (2007). Electromyography of the buccal musculature of octopus (*Octopus bimaculoides*): A test of the function of the muscle articulation in support and movement. J. Exp. Biol..

[48] Roscian M., Souquet L., Herrel A., Uyeno T., Adriaens D., De Kegel B., Rouget I. (2023). Comparative anatomy and functional implications of variation in the buccal mass in coleoid cephalopods. J. Morphol..

[49] Baggett H., Doran G.A. (1971). A structural and functional classification of mammalian tongues. J. Mammal..

[50] Abd-El-Malek S. (1955). The part played by the tongue in mastication and deglutition. J. Anat..

[51] Stone M., Lundberg A. (1996). Three-dimensional tongue surface shapes of English consonants and vowels. J. Acoust. Soc. Am..

[52] Gick B., Allen B., Roewer-Després F., Stavness I. (2017). Speaking tongues are actively braced. J. Speech Lang. Hear. Res..

[53] Perrier P., Payan Y., Zandipour M., Perkell J. (2003). Influences of tongue biomechanics on speech movements during the production of velar stop consonants: A modeling study. J. Acoust. Soc. Am..

[54] Kennedy E.B.L., Buresch K.C., Boinapally P., Hanlon R.T. (2020). Octopus arms exhibit exceptional flexibility. Sci. Rep..

[55] Zullo L., Di Clemente A., Maiole F. (2022). How octopus arm muscle contractile properties and anatomical organization contribute to arm functional specialization. J. Exp. Biol..

[56] Zobek C.M., Porter L.M., Verhulst C., Hostnik E., Aitken-Palmer C., Holliday C.M. (2025). 3D Muscle Architecture of the Tongue of the White-bellied Pangolin (*Phataginus tricuspis*) Reveals a Muscular Hydrostat. Integr. Comp. Biol..

[57] Osuna-Mascaró A.J., Auersperg A.M.I. (2026). Flexible use of a multi-purpose tool by a cow. Curr. Biol..

[58] Drushel R.F., Neustadter D.M., Shallenberger L.L., Crago P.E., Chiel H.J. (1997). The kinematics of swallowing in the buccal mass of *Aplysia californica*. J. Exp. Biol..

[59] Golowasch J., Goldman M.S., Abbott L.F., Marder E. (2002). Failure of Averaging in the Construction of a Conductance-Based Neuron Model. J. Neurophysiol..

[60] Blümel M., Guschlbauer C., Hooper S.L., Büschges A. (2012). Using individual-muscle specific instead of across-muscle mean data halves muscle simulation error. Biol. Cybern..

[61] Beer R.D., Chiel H.J., Gallagher J.C. (1999). Evolution and analysis of model CPGs for walking: II. General principles and individual variability. J. Comput. Neurosci..

[62] Hurwitz I., Susswein A.J. (1992). Adaptation of Feeding Sequences in *Aplysia oculifera* to Changes in the Load and Width of Food. J. Exp. Biol..

[63] Steele C.M., Van Lieshout P.H.H.M. (2004). Influence of bolus consistency on lingual behaviors in sequential swallowing. Dysphagia.

[64] Rohatgi A. (2022). WebPlotDigitizer. https://apps.automeris.io/wpd4/.

[65] Neustadter D.M., Drushel R.F., Chiel H.J. (2002). Kinematics of the buccal mass during swallowing based on magnetic resonance imaging in intact, behaving *Aplysia californica*. J. Exp. Biol..

[66] Aboshosha S., Zahran O., Dessouky M.I., Abd El-Samie F.E. (2019). Resolution and quality enhancement of images using interpolation and contrast limited adaptive histogram equalization. Multimed. Tools Appl..

[67] Sun X., Kerschen G., Cheng L. (2023). Geometrical nonlinearities in a curved cantilever beam: a condensation model and inertia-induced nonlinear features. Nonlinear Dyn..

[68] Taylor C.R. (2016). Finite Difference Coefficient Calculator. https://web.media.mit.edu/%7Ecrtaylor/calculator.html.

[69] Broyden C.G. (1965). A class of methods for solving nonlinear simultaneous equations. Math. Comput..

